# Teaching Principles of Medical Innovation and Entrepreneurship Through Hackathons: Case Study and Qualitative Analysis

**DOI:** 10.2196/43916

**Published:** 2023-02-24

**Authors:** Carl Preiksaitis, John R Dayton, Rana Kabeer, Gabrielle Bunney, Milana Boukhman

**Affiliations:** 1 Department of Emergency Medicine Stanford University School of Medicine Palo Alto, CA United States

**Keywords:** hackathon, innovation, entrepreneurship, medical education, gamification, curriculum, biodesign, emergency medicine, health care innovation, medical innovation, training, design, implementation, development, physician, educational

## Abstract

**Background:**

Innovation and entrepreneurship training are increasingly recognized as being important in medical education. However, the lack of faculty comfort with the instruction of these concepts as well as limited scholarly recognition for this work has limited the implementation of curricula focused on these skills. Furthermore, this lack of familiarity limits the inclusion of practicing physicians in health care innovation, where their experience is valuable. Hackathons are intense innovation competitions that use gamification principles to increase comfort with creative thinking, problem-solving, and interpersonal collaboration, but they require further exploration in medical innovation.

**Objective:**

To address this, we aimed to design, implement, and evaluate a health care hackathon with 2 main goals: to improve emergency physician familiarity with the principles of health care innovation and entrepreneurship and to develop innovative solutions to 3 discrete problems facing emergency medicine physicians and patients.

**Methods:**

We used previously described practices for conducting hackathons to develop and implement our hackathon (HackED!). We partnered with the American College of Emergency Physicians, the Stanford School of Biodesign, and the Institute of Design at Stanford (d.school) to lend institutional support and expertise in health care innovation to our event. We determined a location, time frame, and logistics for the competition and settled on 3 use cases for teams to work on. We planned to explore the learning experience of participants within a pragmatic paradigm and complete an abductive thematic analysis using data from a variety of sources.

**Results:**

HackED! took place from October 1-3, 2022. In all, 3 teams developed novel solutions to each of the use cases. Our investigation into the educational experience of participants suggested that the event was valuable and uncovered themes suggesting that the learning experience could be understood within a framework from entrepreneurship education not previously described in relation to hackathons.

**Conclusions:**

Health care hackathons appear to be a viable method of increasing physician experience with innovation and entrepreneurship principles and addressing complex problems in health care. Hackathons should be considered as part of educational programs that focus on these concepts.

## Introduction

Given the rapid pace of societal and technological changes and the growing complexity of the health care sector, medical education is increasingly focused on skills that will improve the provision of high-value, quality patient care [1]. Innovation, interprofessional collaboration, and entrepreneurship are recognized as critical skills for training physicians to address the challenges of health care in the 21st century. These skills have been incorporated into medical education at the undergraduate and graduate levels [2,3]. The importance of teaching health-systems science is often described as the “third pillar of medical education” [4].

Medical education did not traditionally teach principles of quality improvement, interprofessional collaboration, and health care innovation [5]. Education in this area has improved with the incorporation of health-systems science content into the Association of American Medical College’s Entrustable Professional Activities and Accreditation Council for Graduate Medical Education Milestones [2,3]. However, current curricula may not equip physicians with the innovative strategies needed to address larger and more complex health care problems [6-8]. Several medical schools now include innovation and entrepreneurship curricula that draw on techniques from business and design to develop approaches to solving challenging health care problems [8]. It should be noted that entrepreneurship in this context refers to considering the commercial viability of a solution and is strongly connected to evaluating the feasibility of an innovation [7]. The lack of faculty comfort with the principles of health care innovation and entrepreneurship is an identified barrier to the expansion of these programs [7]. Furthermore, the lack of exposure to these curricula among postgraduate physicians may limit their potential to address systems-level problems uncovered in practice. Despite this need, few programs exist that address continuing professional development in this area, and there is a need for an educational intervention to address this gap [5,8].

A hackathon, a portmanteau of the terms “hack” and “marathon,” is an intense competition where individuals or teams seek to develop novel solutions to challenging problems over a short time period [9]. Hackathons have their origins in the fields of computer science and engineering but more recently have been described as a method of innovation in health care that provides an educational opportunity for all participants [10]. Hackathons are based on the principles of gamification, which refers to the use of game elements (teams, time limits, and prizes) in nongame contexts [11]. Gamification is growing in popularity in medical education, but a complete understanding of the learning experience in gamified activities is still being described [11]. To our knowledge, the use of a hackathon as a method of increasing emergency physician knowledge of the principles of health care innovation and entrepreneurship has not been described.

We aimed to design, implement, and evaluate a health care hackathon with two main goals:

1. To improve emergency physician familiarity with the principles of health care innovation and entrepreneurship
2. To develop innovative solutions to 3 discrete problems facing emergency medicine (EM) physicians and patients

## Methods

### Development of the Hackathon

We, a team of innovation-focused physicians from Stanford’s Department of Emergency Medicine, used the best practices for health care hackathons described by Silver et al [10] in 2016 to guide the development and implementation of our hackathon. Our first task was to identify internal and external stakeholders, explain our vision, and recruit needed support. The American College of Emergency Physicians (ACEP) had previously hosted innovation events at their annual Scientific Assembly, and they had expressed interest in hosting a similar event in the future. We partnered with them to conduct the event during the ACEP Scientific Assembly in San Francisco, California (October 1-3, 2022). We decided on 2 and a half days for the duration of the event, as we did not have access to a space continuously and wanted to allow those in our event the opportunity to be involved with other aspects of the assembly.

Several of the team members previously worked on the Stanford Emergency Medicine Innovations Symposium (StEMI X) [12] and are fellows in the Stanford Emergency Medicine Innovation and Design Fellowship. These members paired their clinical knowledge as practicing emergency providers with previous experience developing innovation competitions to help design this event. We recognized the need for further expertise in design thinking and the business side of innovation, so we partnered with faculty from the Stanford Byers Center for Biodesign [13], a training program designed for health technology innovators. This completed the assembly of our team, all aligned in the development of a successful event, but with unique perspectives: ACEP as a large professional organization representing an interest in developing innovation within the field of EM; Stanford’s Biodesign School contributing academic and industry experience; and the Stanford Emergency Medicine Innovation and Design Fellows integrating the perspectives of these 2 organizations.

Through collaboration using weekly videoconference meetings and asynchronous Slack (Slack Technologies) discussions, we drew on professional experience in EM and health care innovation and arrived at 3 use cases for the teams to work on. These were as follows:

1. Deciding how to use data from personal wearable technology (heart-rate monitors, step-counters, etc) in the emergency or acute care setting
2. Determining how EM can integrate “hospital at home,” where patients receive inpatient-level care through remote monitoring in their home, into our practice
3. Addressing how health care surveillance tools can be used to identify patterns of disease and improve care for patients in the emergency department

These cases were purposefully nonspecific, selected to be relevant to emergency physicians, and included emerging topics without clearly defined solutions. We wished to encourage and motivate individuals to participate by allowing teams to select their own specific problem and making these problems relevant to EM. We focused on the ideation part of the process. Teams were expected to develop an appealing pitch deck for a concept that could be prototyped in the future. We did not want to limit participants to those who had technical skills to develop a working model of their solution.

As a group, we settled on rules and developed a web-based registration form so that participants could select which use case they would like to work on. Advertisements were sent with registration materials 2 weeks before the ACEP Scientific Assembly (Multimedia Appendix 1). Since our target audience was physicians, we paired teams with coaches who had previous experience in health care innovation or biodesign. To further equip participants with the skills necessary to address their designated problems, we recruited a diverse group of speakers to give short presentations on health care innovation topics over the course of the hackathon. These talks were largely informed by content from the Institute of Design at Stanford, also known as the d.school [14], and School of Biodesign [13]. We planned a pitch competition during the final day of the event and recruited a group of judges in leadership positions in EM and health care innovation. The winning prize was free consultation with the Stanford Emergency Medicine Partnership Program [15], an organization of Stanford health care providers who provide consulting services for entrepreneurs in the health care innovation space.

### Study Design

Previous research on health care hackathons has called for additional scholarship that focuses on the use of these events for medical education. Therefore, we designed a study to explore the educational experience of participants [9,10,16]. Our aim in this analysis was to create useful knowledge for the development of future hackathons in this space. With this goal in mind, we elected to conduct this research in a pragmatic paradigm with an abductive methodology. Unlike inductive research, aimed at building theory from interpretive methods, or deductive research, which often aims to objectively test theory, abductive research aims to find a middle ground, with equal engagement with empirical data and existing theory [17]. Rooted in the philosophy of pragmatism, abductive research aims to find the most logical solution and useful explanation for phenomena.

### Data Collection

We planned to gather study data from a variety of sources: direct observation with field notes, informal interviews, web-based documents and communications, and a qualitative questionnaire. We adapted our questionnaire from content previously used by one of the authors to evaluate hackathons as a pedagogical tool for medical students studying population health [18]. A qualitative survey was used in that investigation, and we elected to do the same to allow for a more comprehensive description than a quantitative survey can provide. As others have described, web-based qualitative surveys are usually less burdensome for participants than face-to-face methods, and we anticipated that the considerable time commitment to the hackathon would be a barrier to recruiting participants for interviews [19]. Our survey adaptation was guided by web-based qualitative survey methodology: questions were designed to be open, concise, and unambiguous, and we aimed to keep the survey short to minimize participant fatigue [19]. To optimize content and internal structure evidence, we adapted this survey using an iterative editing approach. The instrument was extensively tested by all the authors for survey functionality, matching of item content to construct, optimal phrasing, and quality control. The survey was piloted within the author group and pilot results were cross-checked for consistency, providing some evidence of response process validity. The survey was distributed to participants by email using the Qualtrics Survey Tool (Qualtrics, Inc) as well as the hackathon team Slack channels. Consent information documenting risks and benefits of participation in the research was distributed with the survey, and completion implied voluntary, informed consent.

Direct observation and informal interviews were performed in the field by one researcher (CP), and detailed field notes, memos, and a reflexivity journal were kept. The researcher’s presence and purpose of conducting observations was made known to all participants. The participants were informed that no identifying information would be documented. Informal interviews were conducted during the hackathon by CP, and the researcher received assent from participants before questions were asked.

### Ethical Considerations

The Stanford University Institutional Review Board deemed this research exempt (IRB 67403).

### Reflexivity

All the authors are EM physicians. CP is a medical education scholarship fellow currently pursuing a master’s degree in medical education, which includes formal training in qualitative research methods. JRD and GB are innovation fellows. MBT is a professor of EM, and RK is a chief EM resident. CP, RK, and JRD identify as male. MBT and GB identify as female. CP, JRD, GB, and MBT delivered educational lectures at the hackathon. JRD, GB, and RK were involved in the development and implementation of the Stanford Emergency Medicine Innovations Symposium and the hackathon.

### Data Analysis

One researcher (CP) evaluated our data using an abductive thematic analysis based on Thompson’s [17] approach. This method draws from the tradition of Braun and Clarke’s [20] reflexive thematic analysis, which centers on the researchers’ role in knowledge production, rather than “coding reliability” approaches, which often use multiple coders and aim for “reliable” or “accurate” coding. Based on our pragmatic paradigm, the subjective nature of a single researcher’s analysis was acceptable to achieve our goal of a logical and useful explanation of the learning experience.

This process was aided by NVivo software (version 1.7; QSR International, Inc). The researcher familiarized himself with the collected study data and generated initial codes. He then reviewed the codes to develop themes. The next step was theorizing, the process of explaining the relationships between themes and data. In keeping with an abductive thematic analysis, “clustering and explanation of themes [was] guided, but not determined by existing theoretical understanding” [17]. CP reviewed the themes in the context of theoretical knowledge and frameworks described in the medical education literature; however, a suitable model was not uncovered. Given that this was an exercise in innovation and entrepreneurship, the search was expanded to include educational literature in these fields. A framework for practice-based entrepreneurship education described by Neck et al [21] was uncovered that provided insight into the developed themes, and a reanalysis of the data sensitized by this framework was completed [21].

## Results

### Implementation

HackED! took place between October 1-3, 2022. Based on registration preferences, individuals were assigned to teams for each use case (3 total). At the start of the event, our preassigned teams were reorganized to accommodate the difference between participants who registered and those who showed up. Each team ended up with 4 core team members who completed the event from start to finish. During the conference, participants met at a dedicated space of the exhibition hall equipped with 4 long tables situated with 2 on each side of a small stage. Each team received a whiteboard, pens, and erasers, and participants were instructed to bring their own laptop or smart device. Each team was provided a dedicated Slack channel to facilitate communication within teams when they were not all gathered in the hackathon space.

The first day ran from 11:00 AM to 3:30 PM with a 1-hour lunch break and four 15-minute lectures. Lecture topics from the first day included innovation in health, needs assessment, design thinking, and considerations for advising or investing in health care start-ups (Multimedia Appendix 1). The second day ran from 9:00 AM to 3:30 PM again with a 1-hour lunch break and included lectures on securing funding and valuation, missing data, application testing, and artificial intelligence. During the second day, several registrants that had been delayed for the first day joined teams. The final day ran from 9:00 AM to 12:30 PM, and the pitch competition occurred between 1:30 and 3:30 PM. Lectures before the pitch competition were on applying the EM mindset to product management and innovation through experience.

Each of the teams delivered pitches to the panel of judges, who were physician leaders, accomplished innovators, informaticists, and technical experts. A final winner was selected based on feasibility and viability, impact, and progress on a solution.

### Description of the Study Sample

In all, 12 participants completed the hackathon from start to finish. Participants identified as physicians (n=9), engineers (n=2), entrepreneurs (n=2), and user-experience designers (n=1). Some identified as multiple roles: 1 engineer/entrepreneur and 1 physician/entrepreneur. For physicians, their clinical experience ranged from 3-31 years in a variety of different clinical settings.

### Learning Experience of the Participants

#### Framework

Neck et al’s [21] formulation of entrepreneurship education requires “a practice-based approach as a model of learning to support entrepreneurial action.” This framework is based in Billet’s [22] conception of practice theory, which postulates that learning activities can “generate richer understanding about practice, but from and through practice, not on behalf of it.” Neck et al [21] describe 5 specific practices in entrepreneurship education: practice of play, practice of empathy, practice of creation, practice of experimentation, and practice of reflection.

#### Practice of Play

This practice focuses on imaginative thinking, games, and competition to develop innovative ways of being entrepreneurial. Hackathons in general are gamified. They are competitions with prizes and time limits and are often team based. Several participants commented on their enjoyment of the competitive nature of the event and indicated that this led to greater enthusiasm for participation.

#### Practice of Empathy

This practice is characterized by the development of skill in feeling and understanding the perspectives of others. Participants were observed to consider needs from a variety of different perspectives: patients, financers, physicians, and insurance companies. As one participant remarked, “there’s a lot to consider...and what might be good for the patient might not be good business.” Participants also appreciated the difference in perspective others from the group shared: “I never had the opportunity to sit down with an engineer and a businessman, I always approach problems from the physician side.” “I reacted to your experience as an ED [emergency department] doc...it helped me understand the physician and patient experience more clearly.” Participants were seen to consider a variety of different perspectives, which was central to the practice of empathy.

#### Practice of Creation

This practice is informed by effectuation theory, which focuses on producing something of value with the resources at hand, even if other resources may be more desirable [23]. Several teams initially were challenged with the limited resources available, and they approached this difficulty in a variety of different ways. The hospital-at-home team felt they had a lack of expertise in this area, so they were able to use their professional contacts to identify someone at the conference with experience in this field to briefly consult with them. The wearable device team initially was working on a glucose-monitoring app, but they felt that they did not have enough collective knowledge to completely develop their idea, so they pivoted to developing a physician wellness app, which they had more experience with. The health care surveillance team recruited other EM physicians to join when they needed additional expertise. All of these activities demonstrate taking action with what is available rather than waiting for the perfect opportunity, a core idea in the practice of creation.

#### Practice of Experimentation

This practice in the tradition of entrepreneurship education draws from problem-based learning, evidence-based learning, and sense making [24]. It is the combination of these theories that encourage students to “act, learn from that action, and build the learning into the next iteration” [22]. This practice can also be seen as similar Kolb’s [25] experiential learning cycle, which describes concrete experience, reflective observation, abstract conceptualization, and active experimentation. The open-ended use cases developed for this event required experimentation with a number of potential problems and solutions, observed in the brainstorming process of all groups. Groups developed an idea, experimented with it in a variety of ways, and then either refined their idea or moved to a new concept. Here, interaction with group facilitators appeared to be a valuable method of experimentation. The wearable group was developing a glucose-monitoring idea and they explored the source of funding for this product with the group facilitator, which identified some problems with marketability. Others noted that discussion with the group identified “knowledge gaps” in their development process that led to refinement.

#### Practice of Reflection

This final practice is a metacognitive process to promote deep learning as a result of the other action practices. In entrepreneurial education, this is often facilitated, and although this was not our a priori intention, our qualitative survey encouraged reflection by several participants. When asked about learning experiences from the event, several participants commented on the team dynamics: “[I learned] how to interact with others when I’m not the formal leader,” and “[we] learned to come together to quickly listen to each other [and] generate ideas.” They also highlighted the event was “incredible for networking,” and as one person said, “[I] met incredible people that I never would have met otherwise.” Lecture content was also reflected on as being “valuable,” showing “the process of working through real problems,” and illustrating “design thinking tactics,” as well as, “the mental models one might use to evaluate medical business ideas.”

Others noted that the event would have an effect on future career plans: “I walked away with more clarity on the role I would like to play when working in healthcare innovation,” and, “[it] showed me some avenues to get more involved as a physician.” Overall, participants’ emotional response to the event was positive, commenting, “loved it,” “100% would repeat,” and “it was a joy...deeply satisfying to direct energy to something that could truly make the world a better place.” These data suggest that participants underwent reflective practice on their experience and learning.

### Solutions Developed

The hackathon teams developed a pitch deck describing an idea for an innovative solution to each of the 3 use cases. The wearable health care data team developed “Happiness Rx,” a lifestyle-tracking app designed to combat physician burnout. The app would provide recommendations for ideal shift scheduling, sleep, and nutrition to optimize physician performance and improve mental health. The hospital-at-home team developed “Dorothy.ai,” an app-based measure using validated clinical decision-making tools to screen patients to both determine the safety of discharge as well as the coordination of their expected resources needed at home. The surveillance team developed “ForecastER,” a subscription-based service for hospitals to get real-time maps of disease patterns to help emergency departments and hospitals prepare their staff and resources for potential patient surges. Based on the evaluation of the teams’ pitch, the panel of judges declared “Dorothy.ai” the winner. This solution had the greatest potential to translate into a viable product through continued development.

## Discussion

### Principal Findings

Here, we report on our experience with HackED!, a health care hackathon designed to improve EM physician experience with health care innovation by addressing 3 use cases relevant to EM. In terms of generating solutions to the use cases, the event was a success. We arrived at 3 innovative solutions that addressed the problems laid out for the competition.

Our data also support that the event was meaningful in terms of not only improving participant familiarity with health care innovation but in teaching entrepreneurship within a practice-based model. Health care education has generally focused on medical knowledge and practice, and the methodology used to inform those educational practices may not be effective in a different field. It is telling that we had difficulty capturing the learning experience of this event using educational theory commonly referenced in medical education literature. Health care innovation is more closely related to entrepreneurship as a practice, and thus, it makes sense that our results fit better in a framework from education in that field.

Considering *innovation and entrepreneurship* curricula are of growing interest at both the undergraduate and graduate levels of medical education, the lack of faculty comfort with these concepts as well as the methods of teaching them are of importance [7]. Similarly, in designing future events to teach health care innovation, organizers should be aware of the different educational approaches that may be of relevance to make these events of maximum benefit to participants.

Neck et al’s [21] practice-based approach, including practice of play, practice of empathy, practice of creation, practice of experimentation, and practice of reflection, provides a framework for considering the learning experiences of hackathons. Future organizers of hackathons or other innovation curricula may find this to be a useful framework in considering how participants engage with the event and might include aspects that encourage the development of the described practices.

Our experience demonstrates that a relatively short, competition-based event can have educational value in teaching entrepreneurship and innovation principles. Holding a hackathon may be a way to add to an innovation curriculum or incorporate some innovation experience into medical education at all levels. Through the adaptation of the problem and scope of the event, hackathons could be developed for problems unique to other medical specialties or be used to develop more cross-specialty collaboration.

We plan to repeat this event in 2023 in partnership with ACEP and will draw upon our experience from this endeavor, as well as our new understanding of entrepreneurship education theories, to design our next hackathon in a way that encourages the 5 practices we describe in this paper. We also are exploring ways of continued involvement with teams to develop ideas into viable products and follow-up evaluations to determine the longer-term value of the knowledge gained. Finally, we are considering broader recruitment strategies to further diversify our participants and ways to optimize the timing of this event with the ACEP Scientific Assembly. The optimal size of teams for a hackathon of this type, the advantages of having multiple use cases versus a single use case, and the effects of the diversity of participants on learning are questions we hope to answer in the future.

### Limitations

This report has several limitations. We describe one event with a limited number of participants, and it is likely that this sample only reflects the most enthusiastic participants. Our study was not designed or conducted in a way that objectively evaluates learning experiences, and our inferences regarding learning based on self-reported information and observation were not designed to provide definitive answers about the knowledge gained by participants. Our study also does not provide information about the implementation or durability of this new knowledge. The outcomes seen in this study are not generalizable to a larger group of EM physicians, but we hope that our data inspire further investigation into hackathons as a viable learning modality for health care innovation.

### Conclusion

Skills in health care innovation, interprofessional communication, and entrepreneurship are increasingly recognized as fundamental to tackling the complex health care challenges of the 21st century. These skills can empower health care professionals to lead from within. However, the lack of training in the development of these skills remains a barrier for such engagement and the resulting impact. Although a number of medical institutions are now offering such curricula, their broader adoption is limited by the lack of faculty training in this area. Health care hackathons appear to be one viable method of achieving this aim and could be offered within a continuing professional development program on health care innovation.

## Appendix

Multimedia Appendix 1 Hackathon advertisement, lecture materials, and questionnaires administered to participants and facilitators.

## Abbreviations

ACEP: American College of Emergency Physicians
EM: emergency medicine

## Data Availability

The data sets generated during this study are available from the corresponding author upon reasonable request.
