# Supplementary material for: Teaching Principles of Medical Innovation and Entrepreneurship Through Hackathons: Case Study and Qualitative Analysis
Source: JMIR Med Educ. 2023 Feb 24;9:e43916. doi: 10.2196/43916 (PMC10007000; doi:10.2196/43916)
Supplement: Multimedia Appendix 1 [file mededu_v9i1e43916_app1.pdf]

Help Build the Next Generation of EM Solutions

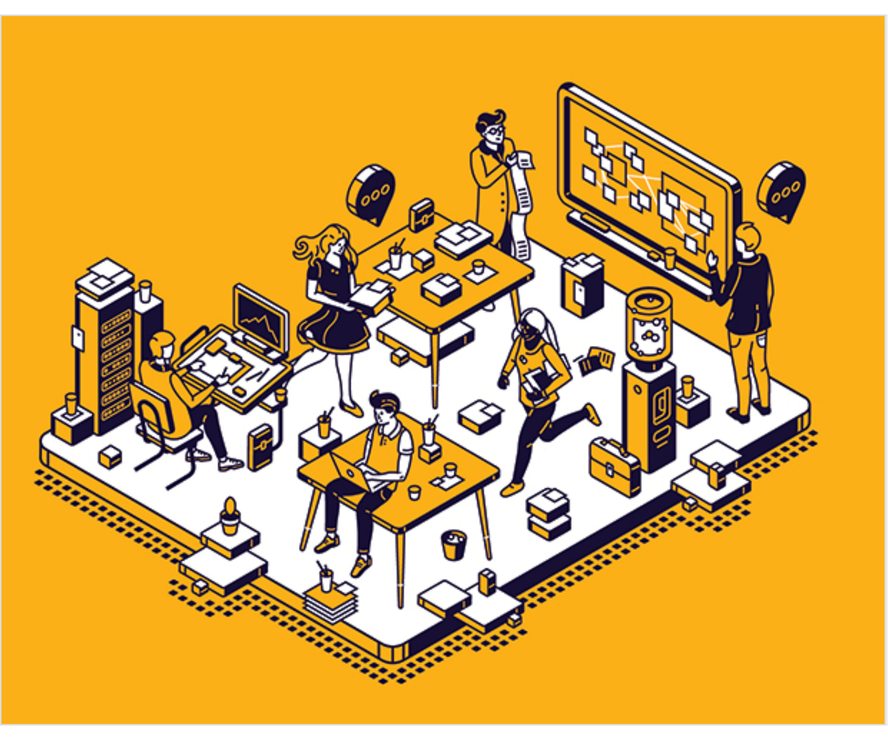

Join us for HackED! - a forum for discovery and innovation at a hackathon event during ACEP22!

Presented in conjunction with Stanford University StEMI X innovators, the ACEP Emergency Medicine Data Institute and ACEP's Health IT Committee, **HackED!** can help solve your most pressing emergency medicine problems.

Harness the power of emergency medicine problem-solving with the technical know-how of proven innovators.

We're near Silicon Valley, the high-tech hub of the San Francisco Bay Area. Let's lean into that proximity and leverage some EM innovation during ACEP22.

Sign Up & Hack Away

HackED! Basics

- Any ACEP22 registered attendees can sign up
- HackED! is **free** for ACEP22 attendees to participate
- Showcased on the floor of the ACEP22 Exhibit Hall at Moscone Convention Center
- Choose one of these use cases to problem-solve (see more below):
  - Wearable Health Data
  - Hospital at Home
  - Health Care Surveillance Tools
- For two-and-a-half days, 12 teams of up to 8 diverse experts will work on impactful, practical EM solutions.
- Concepts and works-in-progress can be viewed each day.
- All ACEP22 attendees can provide feedback as the teams work on their solutions.
- Prizes will be awarded from a panel of judges.

Sign Up & Hack Away

THANK YOU TO OUR 2022 HACKED! SPONSORS

collectivemedical® A PointClickCare Company

belle.ai precision skin health™ | GORDON AND BETTY MOORE FOUNDATION | PA

More About the Use Cases

After signing up for a specific use case, you will be connected with your team and are encouraged to start working on your solution even prior to ACEP22.

Team members, representing subject matter expertise across clinical and technical domains, will receive information that describes the problem, relevant literature, and any resources (such as data or an example app) that may be helpful.

Teams will be self-governed and are responsible for coordinating their workflows and schedules. Coaches will be made available for each team onsite.

Wearable Health Data +

Hospital at Home +

Health Care Surveillance Tools +

More About the Competition

HackED! will be judged by a mix of five physician leaders, innovators, informaticists, and technical experts. On the last day of the hackathon, the judges and audience will hear members of the teams pitch their solutions, which do not have to be a finished product, only compelling and supported.

Judges Criteria

- Feasibility/Viability
- Impact
- Progress

Important Participant Information

- Bring your own devices
- Engagement > Skills
- If your plans change and you cannot attend or cannot fully commit, let ACEP know as soon as possible

HackED! Schedule

All times are Pacific. Schedule is subject to change.

Saturday, October 1

Time	Activity
11:00 am – 11:10 am	Introduction
11:10 am – 11:30 am	Featured speaker: **Jacqueline Shreibati** - "Innovation in Health: Learning from (and beyond) Silicon Valley"
11:30 am – 12:15 pm	Team formation, coaching and discussion
12:15 pm – 12:30 pm	Design speaker: **Josh Makower** - "The Case for Need-Driven Innovation in Health Technology Design"
12:30 pm – 1:30 pm	Lunch break (on your own)
1:30 pm - 1:45 pm	Design speaker: **Milana Boukhman** - "Design Thinking Approach; Brainstorming Primer"
1:45 pm - 2:00 pm	Design speaker: **John Dayton** - "5 T's and 6 P's Emergency Physicians Can Use to Evaluate Pitch Decks for Investing and Advisory Roles"
2:00 pm – 3:30 pm	Problem-solve at workstations

Sunday, October 2

Time	Activity
9:30 am – 9:40 am	Coach check-in
9:40 am – 9:55 am	Design speaker: **James Wall**
9:55 am – 12:15 pm	Problem-solve at workstations
12:15 pm - 12:30 pm	Design speaker: **Carl Preiksaitis** - "Missingness in Medicine: Addressing the Messiness of Healthcare Data"
12:30 pm – 1:30 pm	Lunch break (on your own)
1:30 pm - 1:45 pm	Design speaker: **Kendrah Baker** - "Open Source Healthcare Interoperability Platform: Meld"
1:45 pm - 2:00 pm	Design speaker: **Gabrielle Bunney** - "Applying AI to EM"
2:00 pm – 3:30 pm	Problem-solve at workstations

Monday, October 3

Time	Activity
9:30 am – 9:40 am	Coach check-in
9:40 am - 9:55 am	Design speaker: **Sherman Leung** - "Applying the EM mindset to Product Management"
9:55 am - 10:10 am	Design speaker: **Justin Moore**
10:10 am – 12:30 pm	Problem-solve at workstations
12:30 pm – 1:00 pm	Lunch break (on your own)
1:00 pm – 3:30 pm	Pitch competition – *all ACEP22 attendees are encouraged to watch presentations about the solutions created during HackED!*

Sign Up & Hack Away

PHILADELPHIA, PA

Scientific Assembly PHILADELPHIA, PA 23

Make Plans for ACEP23 in Philadelphia October 9-12, 2023

October 9-12, 2023
We look forward to seeing you there!

Register Today | Book Hotel

Stanford

How would you describe your background? (physician, designer, entrepreneur)

If you are a physician, how many years have you been in practice and how would you describe your practice setting (Academic, community, rural, etc)?

Have you participated in any educational experiences focusing on healthcare innovation before? Please describe.

What did you learn from the hackathon?

Would you continue to pursue and develop your final project idea? Why or why not?

Did this experience inspire you to develop any other innovations? Describe.

Describe the overall experience. How could this experience be improved and used by others?

How satisfied were you with your overall experience?

- Extremely dissatisfied
- Somewhat dissatisfied
- Neither satisfied nor dissatisfied
- Somewhat satisfied
- Extremely satisfied

What is the likelihood of you recommending this experience to others?

- Extremely unlikely
- Somewhat unlikely
- Neither likely nor unlikely
- Somewhat likely
- Extremely likely

→

Powered by Qualtrics

Stanford

Where is the majority of your clinical practice (state or country)?

How would you describe your current practice setting? (Academic, Community, County, Rural, etc)

Have you participated in any educational experiences focusing on healthcare innovation before? Please describe.

What do you feel participants learned from the hackathon?

Describe any challenges you had while facilitating the hackathon.

Describe the overall experience. How could this experience be improved for participants?

How satisfied were you with your overall experience as a facilitator?

- Extremely dissatisfied
- Somewhat dissatisfied
- Neither satisfied nor dissatisfied
- Somewhat satisfied
- Extremely satisfied

How likely are you to recommend participating in a hackathon to a colleague?

- Extremely unlikely
- Somewhat unlikely
- Neither likely nor unlikely
- Somewhat likely
- Extremely likely

→

Powered by Qualtrics

Stanford MEDICINE | Emergency Medicine

d. HASSO PLATTNER Institute of Design at Stanford

Hackathon

Design Thinking Approach: IDEATE
ACEP 2022

STANFORD BYERS CENTER FOR BIODESIGN

Milana Boukhman, MD MBA FACEP
Clinical Professor of Emergency Medicine
Director, BioSecurity and Pandemic Resilience
Department of Emergency Medicine
Stanford Medical School
milanab@Stanford.edu

Design Thinking Approach

STANFORD BYERS CENTER FOR BIODESIGN

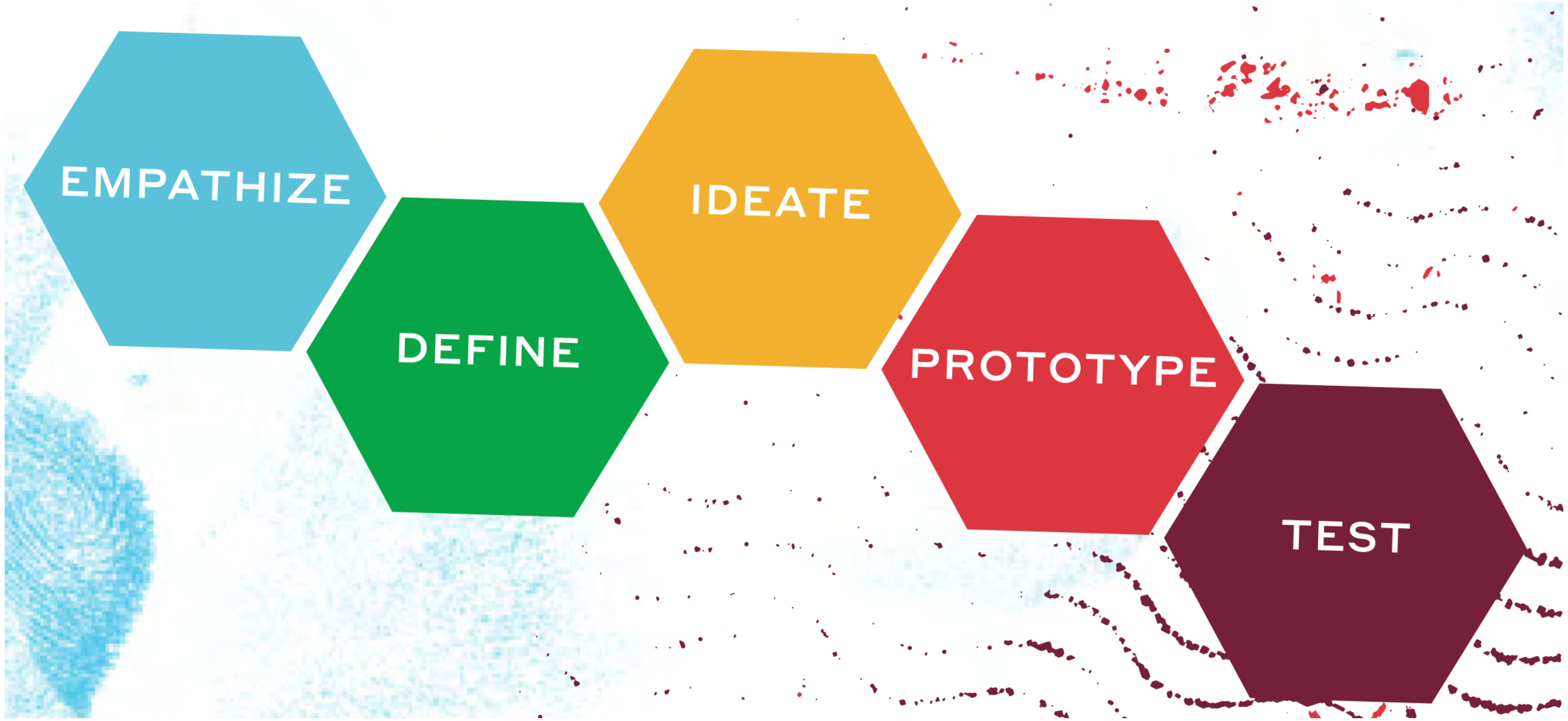

Design Thinking Approach

- **Beginner’s mindset**
  - Don’t judge
  - Question everything
  - Be truly curious
  - Find patterns
  - Listen. Really

- **What? How? Why?**
  - Tool to reach deeper levels of observation
  - Set-up
  - Concrete observations
  - Move to understanding
  - Interpretation

HOW MIGHT WE...

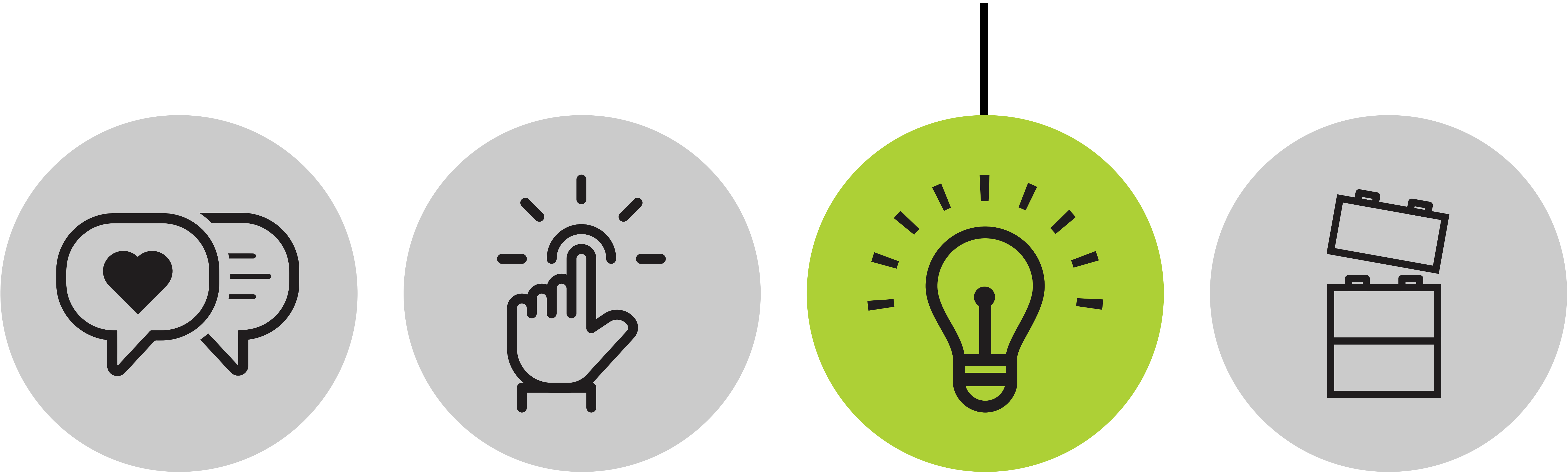

Empathize Synthesize Ideate Prototype

HOW MIGHT WE QUESTIONS

How Might We...

Assumes there's a solution

Assures it's ok whether an idea works or not

Emphasizes collaboration

Source: Stanford d.school

TOOL TO GENERATE IDEAS

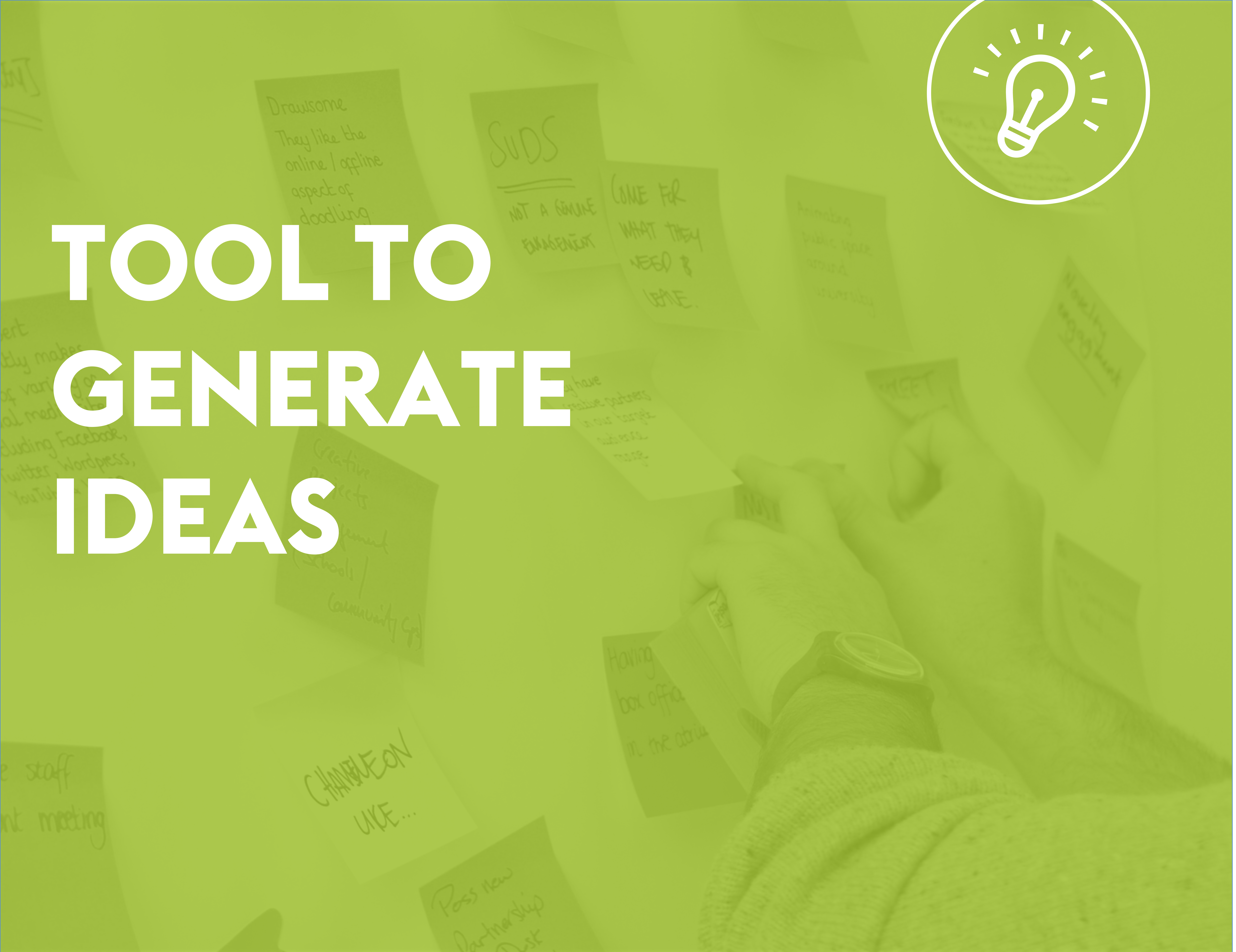

BRAINSTORMING RULES

Idea? Write it down!

- One idea/Post-it
- Legible
- Pictures and words
- Capture enough to be understood later

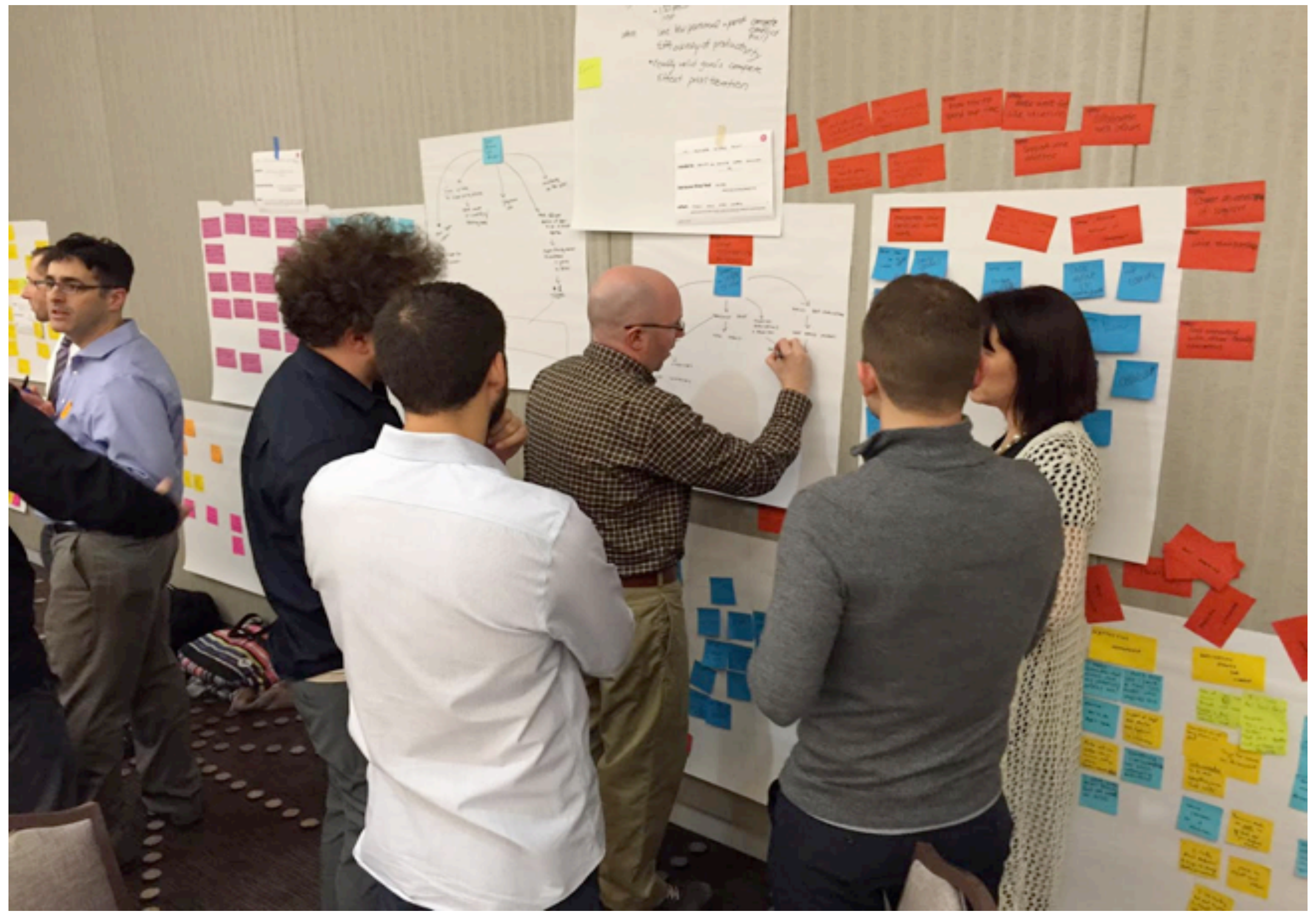

Focus on nouns (ideas), not verbs (needs)

Quantity > Quality

Build on others' ideas: "Yes, and…" + encourage wild ideas

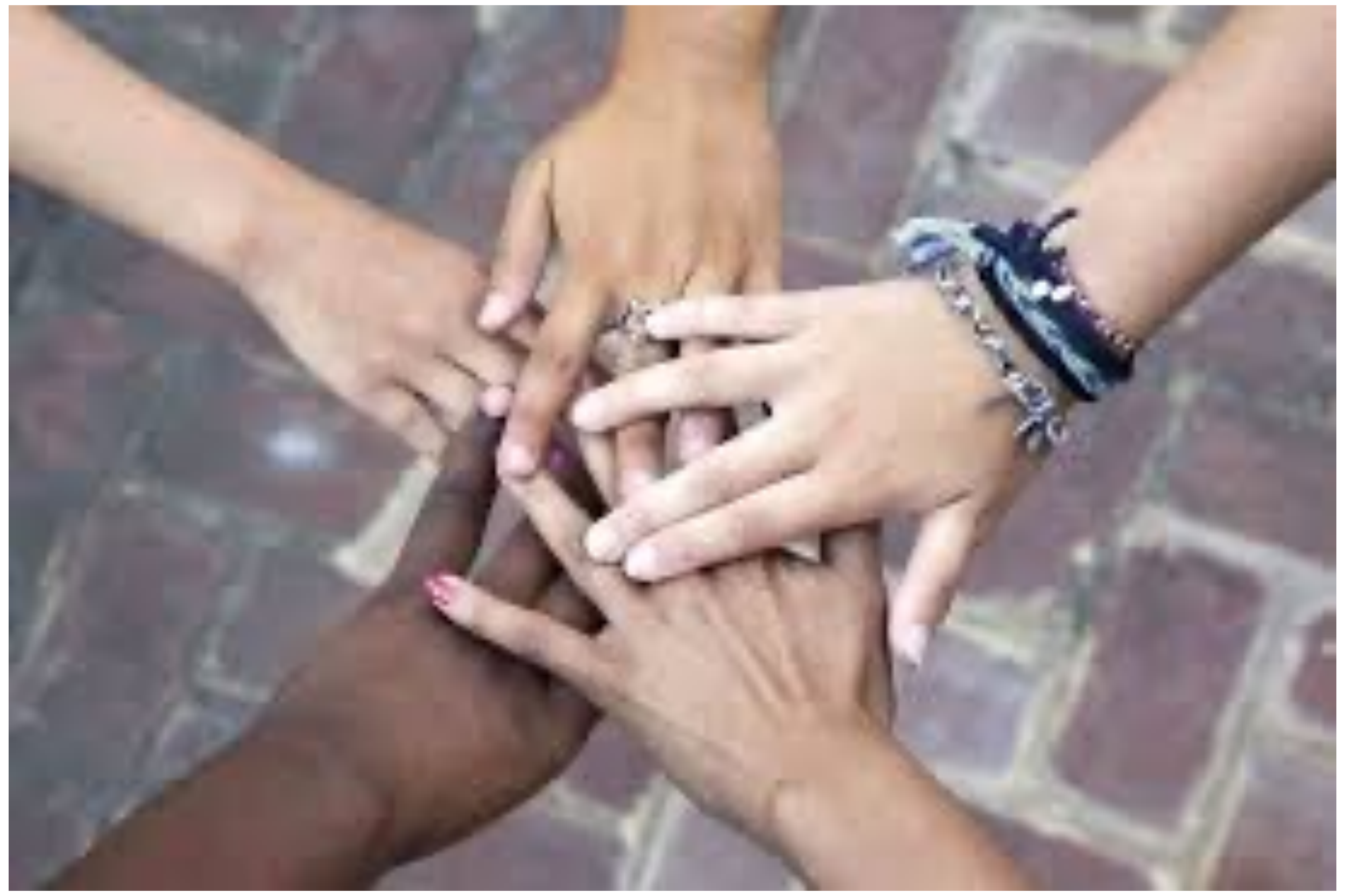

Suspend judgment

Brainstorming Rules

- One Conversation at a time
- Be Visual
- Its not a concept until its written down
- Turn comments into concepts
- Get a facilitator to enforce rules

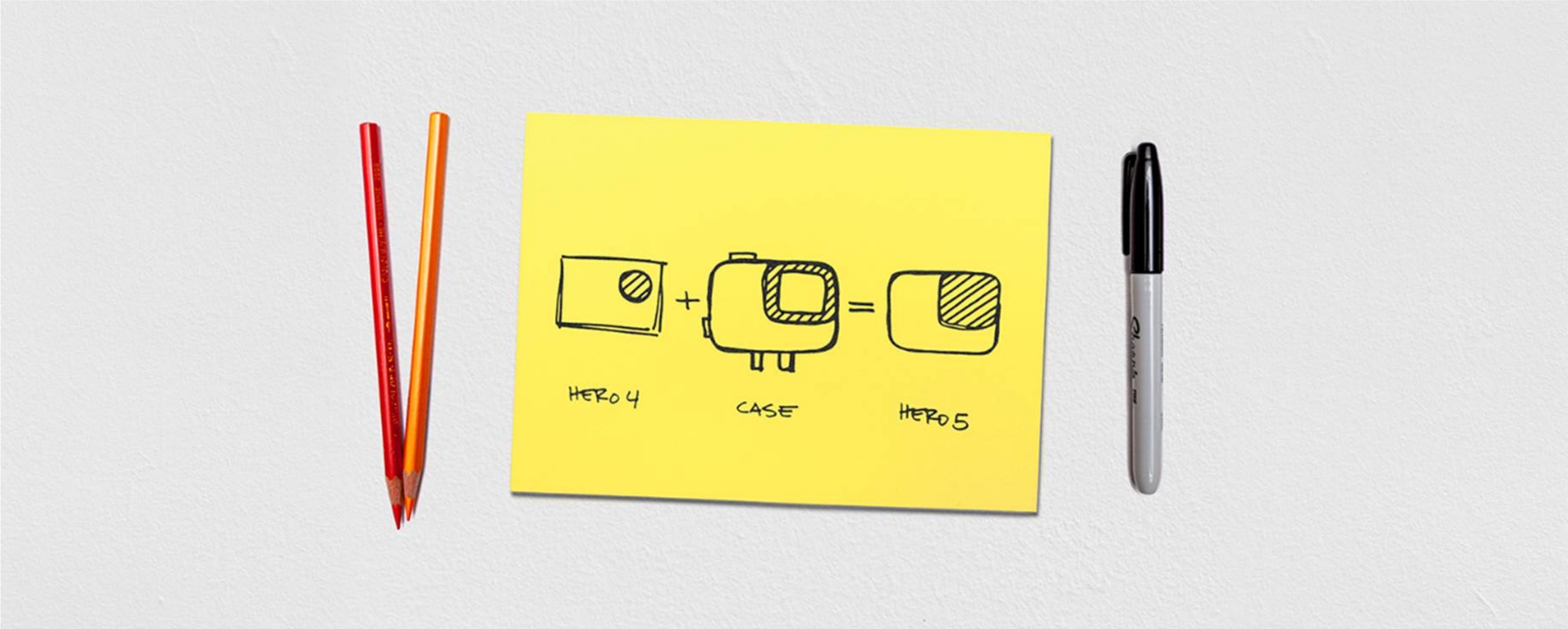

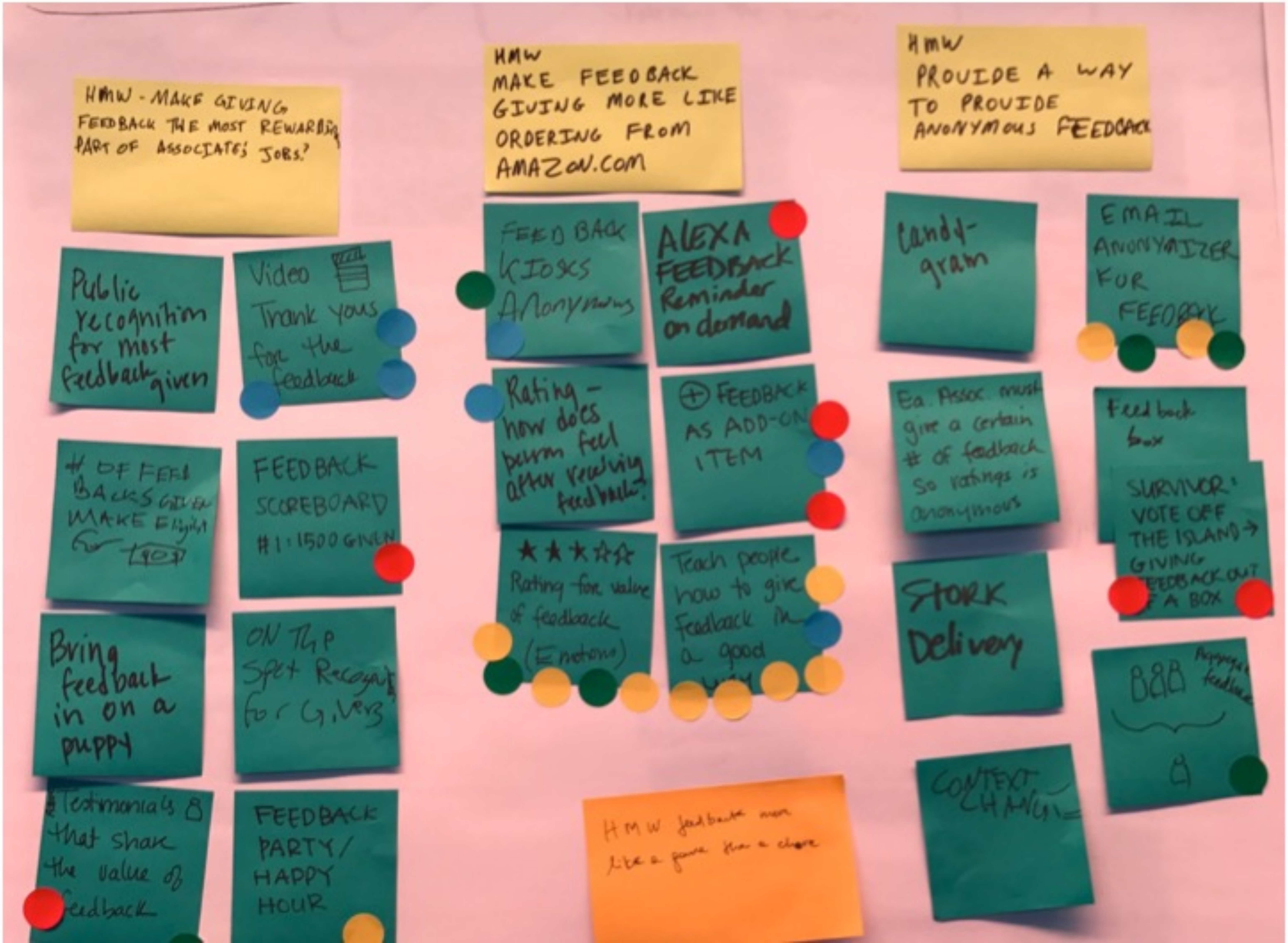

HMW - MAKE GIVING FEEDBACK THE MOST REWARDING PART OF ASSOCIATES' JOBS?
Public recognition for most feedback given
Video Thank yous for the feedback
OF FEEDBACKS GIVEN MAKE
FEEDBACK SCOREBOARD #1: 1500 GIVEN
Bring feedback in on a puppy
ON THE Spot Recognition for Givers
Testimonials that share the value of feedback
FEEDBACK PARTY/ HAPPY HOUR
HMW MAKE FEEDBACK GIVING MORE LIKE ORDERING FROM AMAZON.COM
FEEDBACK KIOSKS Anonymous
ALEXA FEEDBACK Reminder on demand
Rating - how does person feel after receiving feedback?
FEEDBACK AS ADD-ON ITEM
Rating for value of feedback (Emotion)
Teach people how to give feedback in a good way
HMW feedback more like a game than a chore
HMW PROVIDE A WAY TO PROVIDE ANONYMOUS FEEDBACK
Candy-gram
EMAIL ANONYMIZER FOR FEEDBACK
Ea. Assoc must give a certain # of feedback so ratings is anonymous
Feedback box
SURVIVOR: VOTE OFF THE ISLAND → GIVING
STORK Delivery
B&B Aggregate feedback
CONTEXT CHANGE

VOTING + CRITERIA

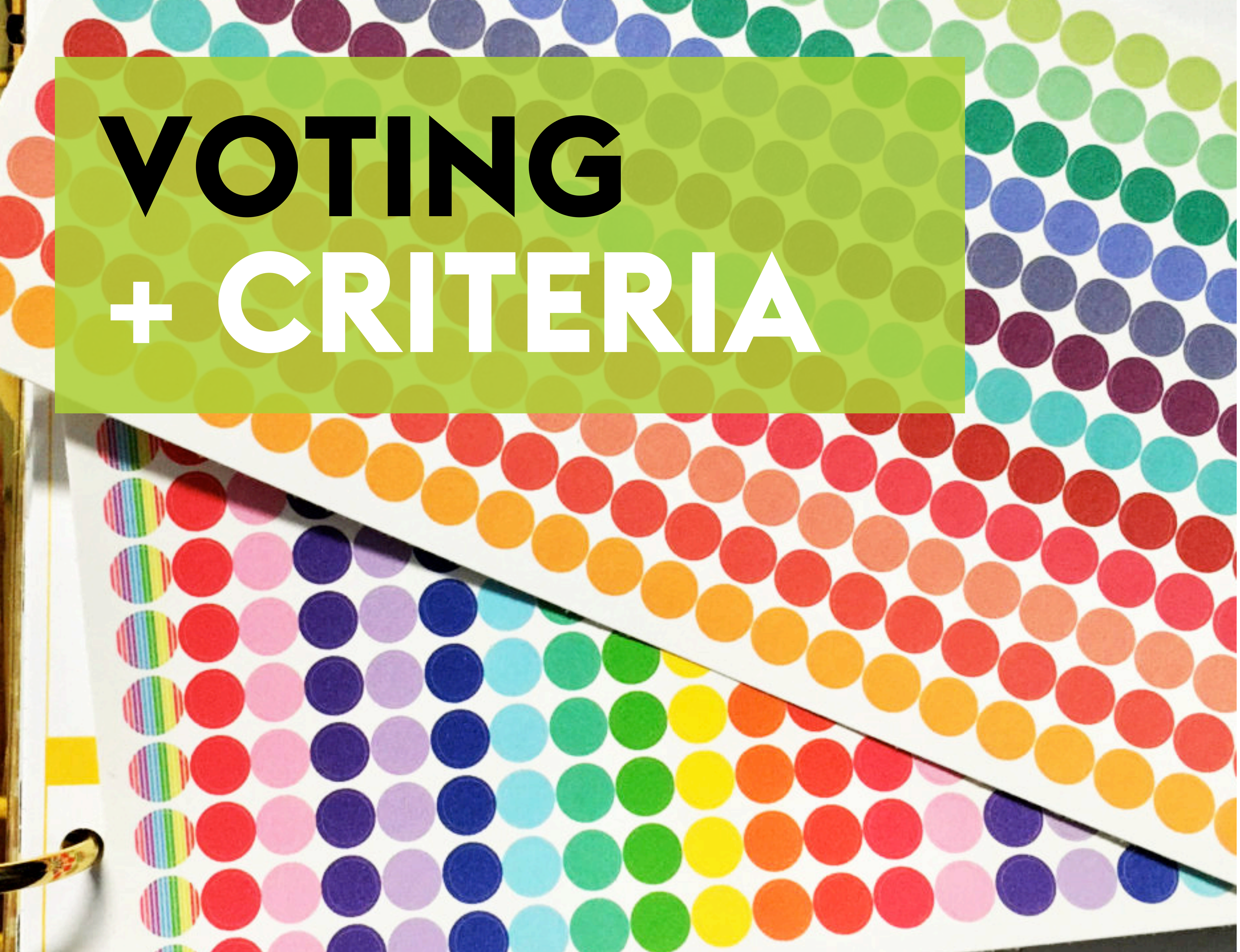

IDEA SELECTION

Vote on the 3 ideas that fit the assignment best

My favorite!
This idea sticks out and excites me.

Journey Map

A tool to capture the story of an **end-to-end** user experience and force **deeper reflection** at each activity

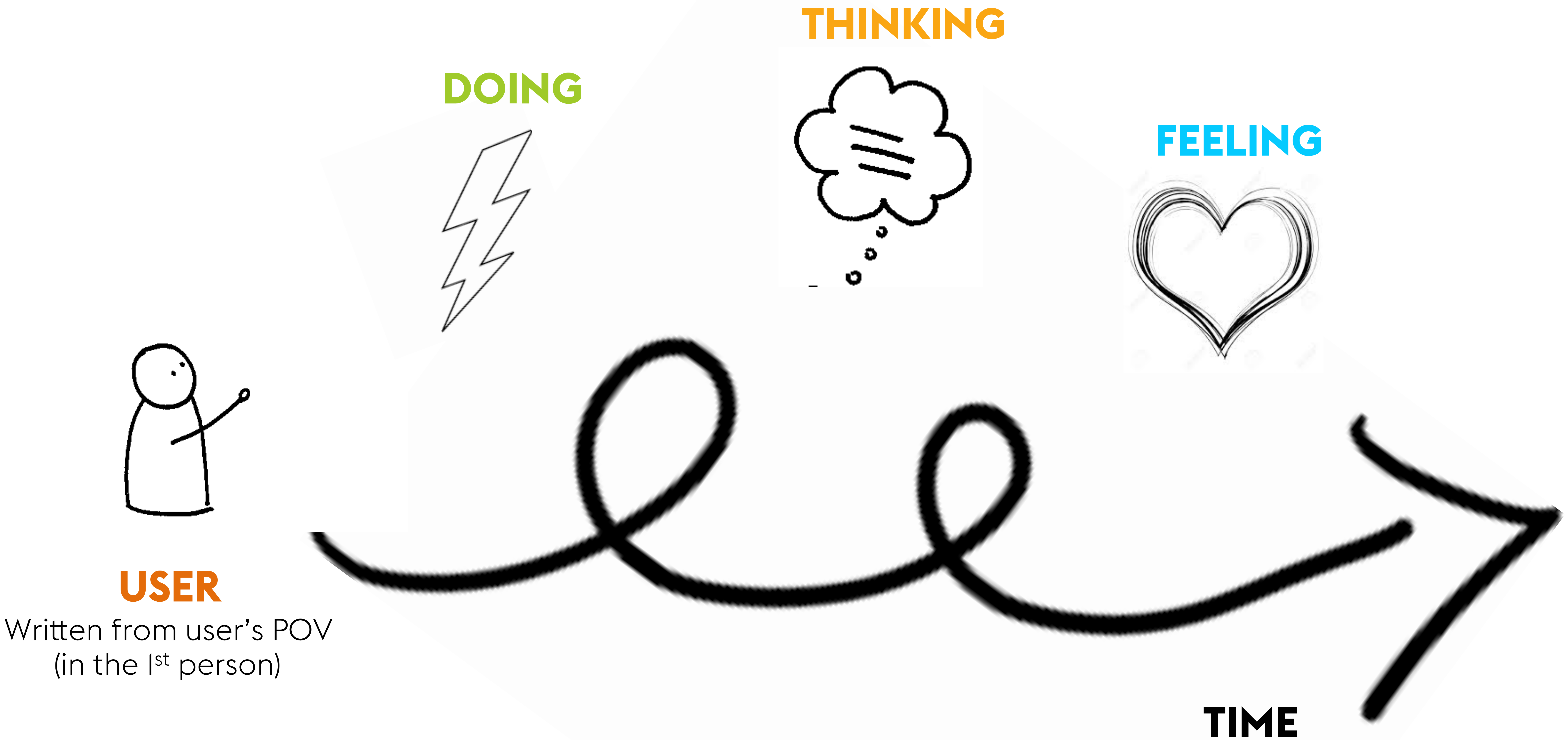

Nurse’s Caregiving Journey

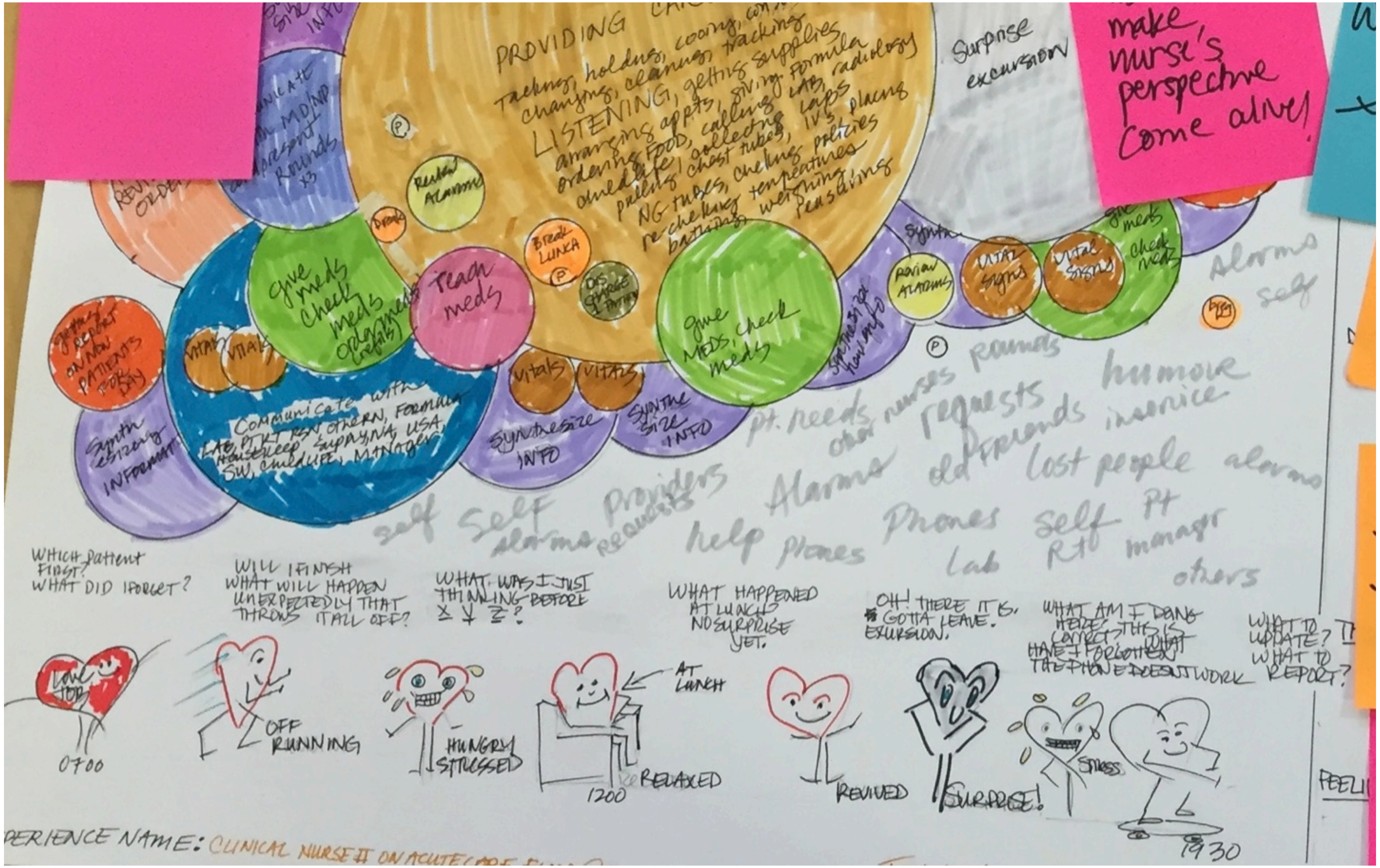

ED Patient Experience

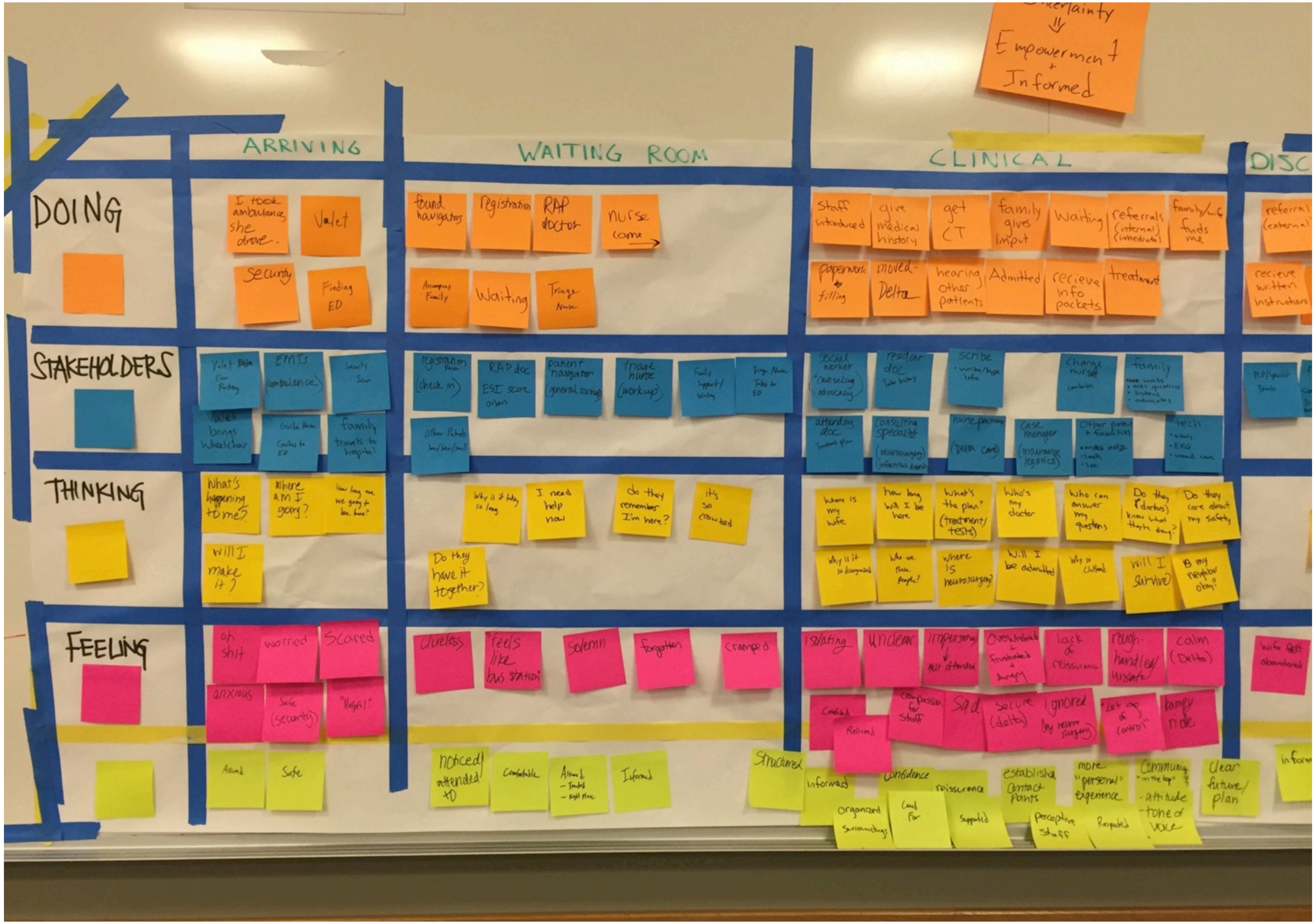

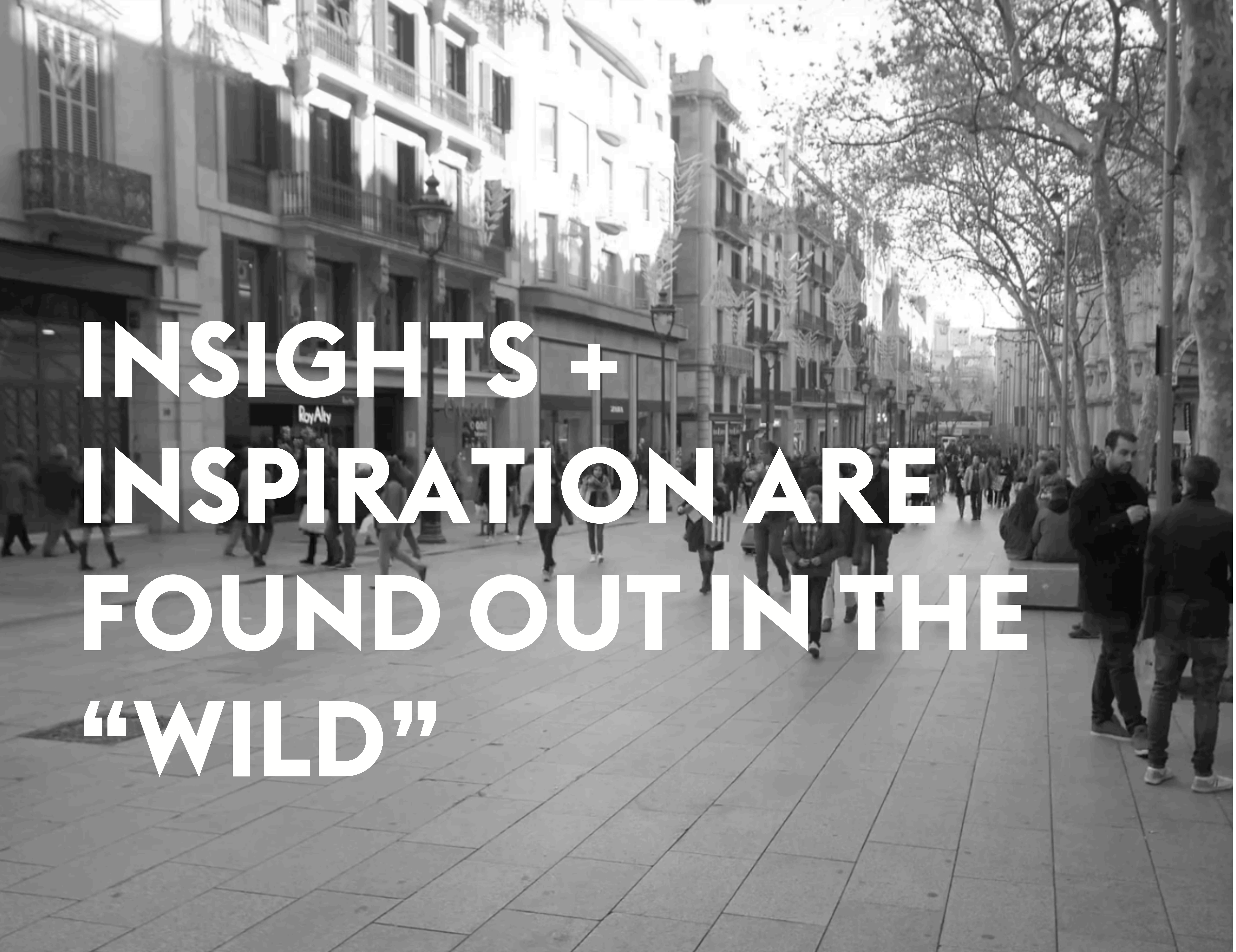

INSIGHTS + INSPIRATION ARE FOUND OUT IN THE "WILD"

STEP ONE

CONDUCT RESEARCH

STEP TWO

GENERATE IDEAS

EMPATHY RESEARCH

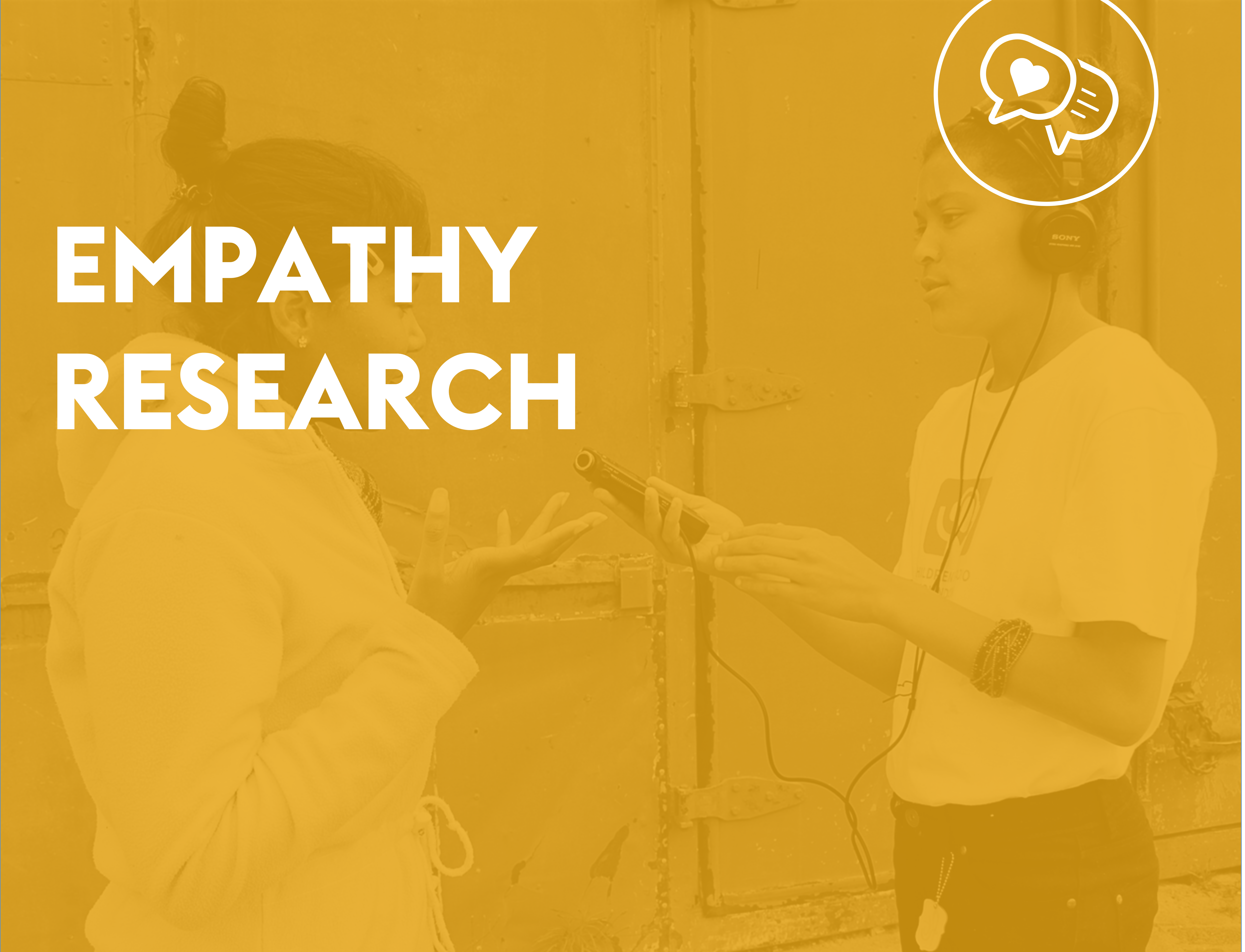

Hackathon Resources

- **Design Thinking Process**

1. Student Guide to Biodesign
2. Biodesign Process Overview
3. Bootcamp: design thinking guide

- **Brainstorming**

1. Coggle
2. Jamboard
3. Miro

- **Slides**

1. SlidesGo
2. Noun Project

Background

- Changes in society and technologies relevant to health are arriving at an unprecedented pace
- A Lancet medical commission of international academic leaders (Frenk et al, 2010) called for **instructional reforms** to promote education that breaks down professional silos while enhancing collaborative and non-hierarchical relationships in effective teams.
- The Lancet commission called for transformative learning to develop leadership attributes and produce **“enlightened change agents”.** This vision requires medical professionals to have knowledge and skills that go beyond the basic and clinical sciences, and which include ability to work collaboratively in **transdisciplinary teams that may include engineers, business developers and policy makers.**
- In medical education, the teaching of these skills is generally limited.
- The design thinking methodology can be deployed to help fill this educational gap. (Brown 2008, Hasso Plattner 2010, van der Grift et al 2016, Nagel et al. 2017, Wolcott & McLaughlin 2020).
- Numerous medical educators have suggested that it can also help address complex challenges in healthcare (Roberts et al. 2016; van de Grift and Kroeze 2016; Gottlieb et al 2017; Nagel et al. 2017; McLaughlin et al. 2019; Wolcott & McLaughlin 2020).
- Design thinking is still an **‘emerging research front’** in medical education (McLaughlin et al. 2019). Given the limited peer-reviewed literature on both the practice and theory pertaining to design thinking, some authors have commented on the challenges of operationalizing teaching these skills and have called for additional scholarship in this area (Brazile et al 2018, Madson 2021).

Applying AI in EM

Presented By Gabrielle Bunney, MD MBA

Innovation and Design Fellow

Stanford Department of Emergency Medicine

No Disclosures

Stanford MEDICINE | Emergency Medicine

Outline

- Defining AI
- Uses of AI in EM practice
- Current limitations of AI

Outline

- Defining AI
- Uses of AI in EM practice
- Current limitations of AI

Framework

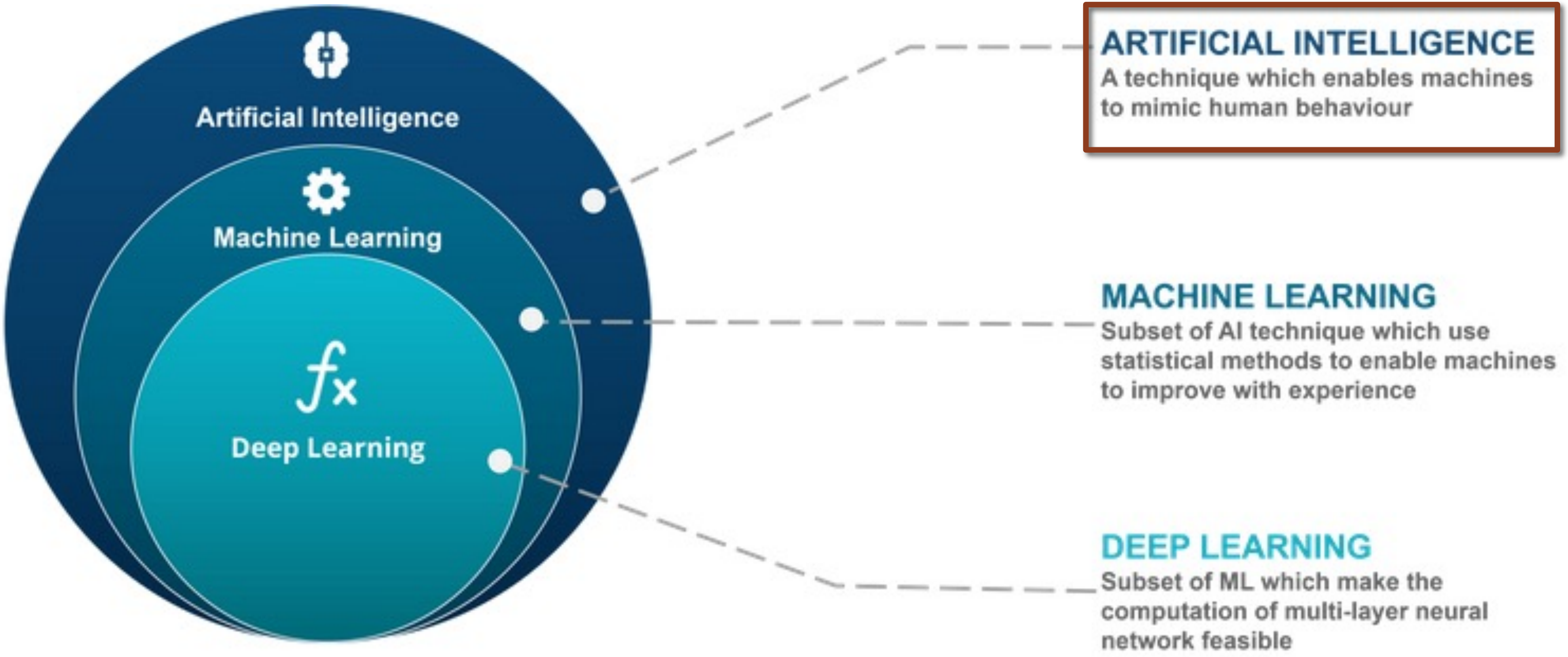

https://www.edureka.co/blog/ai-vs-machine-learning-vs-deep-learning/

Artificial Intelligence

"Broad subset of computer science that simulates human intelligence, including speech recognition, predictive modeling, and problem solving"

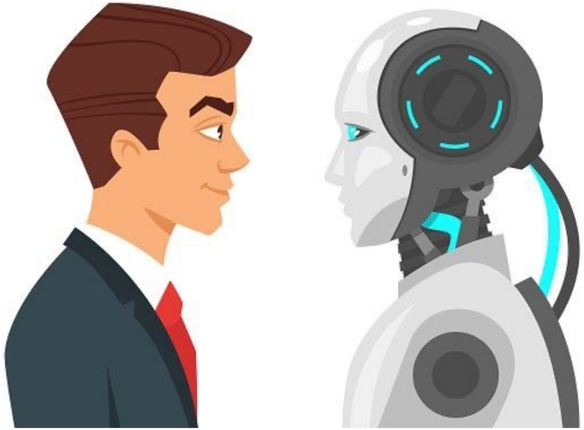

Kirubarajan A, Taher A, Khan S, Masood S. Artificial intelligence in emergency medicine: A scoping review. J Am Coll Emerg Physicians Open. 2020 Nov 7;1(6):1691-1702. doi: 10.1002/emp2.12277. PMID: 33392578; PMCID: PMC7771825.

Framework

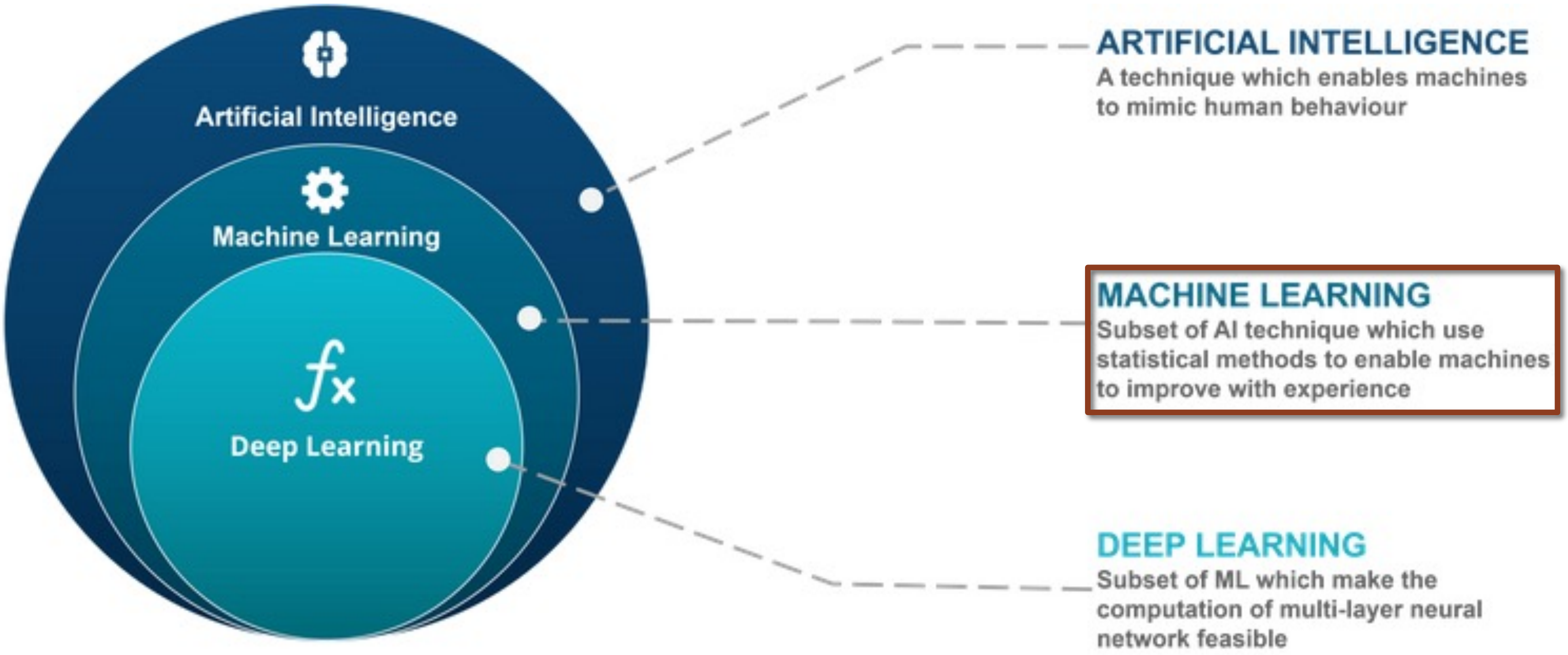

https://www.edureka.co/blog/ai-vs-machine-learning-vs-deep-learning/

Machine Learning

"Computer systems that are able to learn and adapt without following explicit instructions, by using algorithms and statistical models to ***analyze and draw inferences from patterns in data***"

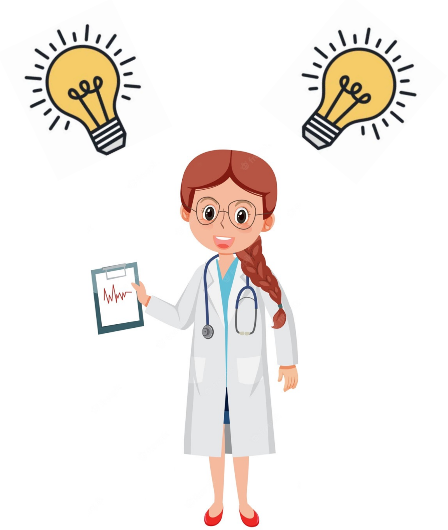

Oxford Languages

Framework

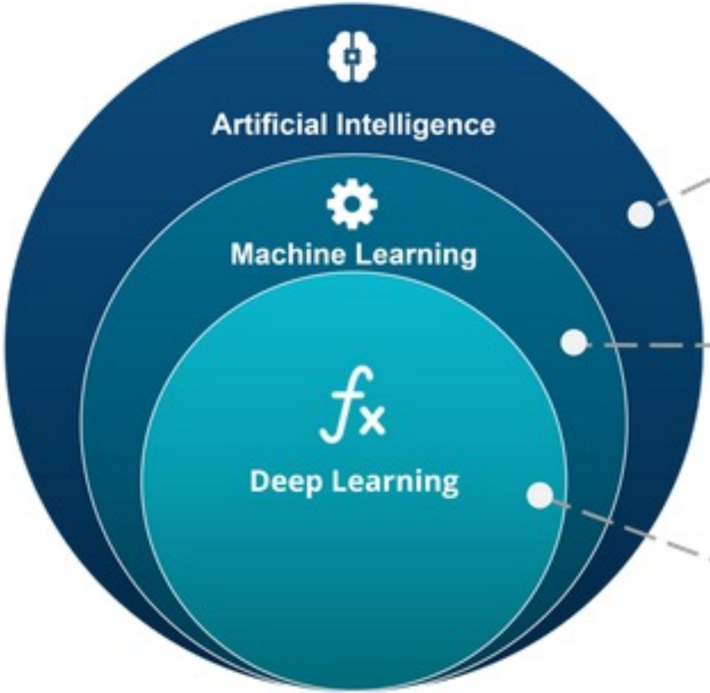

ARTIFICIAL INTELLIGENCE
A technique which enables machines to mimic human behaviour

MACHINE LEARNING
Subset of AI technique which use statistical methods to enable machines to improve with experience

DEEP LEARNING
Subset of ML which make the computation of multi-layer neural network feasible

https://www.edureka.co/blog/ai-vs-machine-learning-vs-deep-learning/

Deep Learning

"A type of ML based on artificial neural networks in which multiple layers of processing are used to extract progressively higher level features from data"

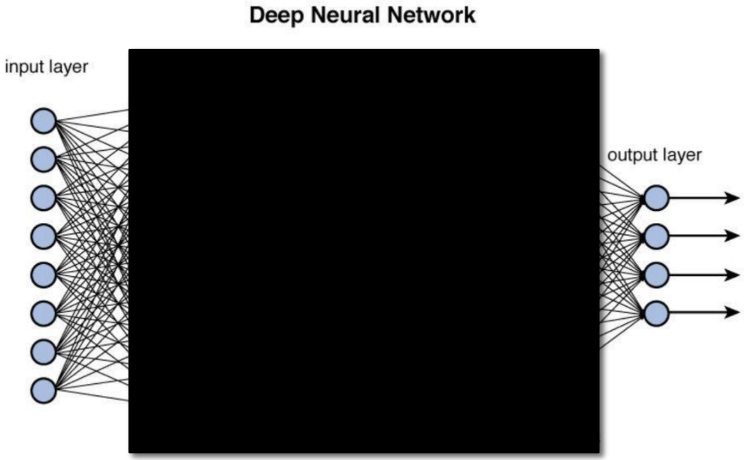

Stanford MEDICINE | Emergency Medicine

Oxford Languages

https://towardsdatascience.com/training-deep-neural-networks-9fdb1964b964

Outline

- Defining AI
- Uses of AI in EM practice
- Current limitations of AI

Prediction

- Technique that allows for processing multiple variables large volumes of data
- Predictions:
  - Admission
  - Complication rates
  - Mortality

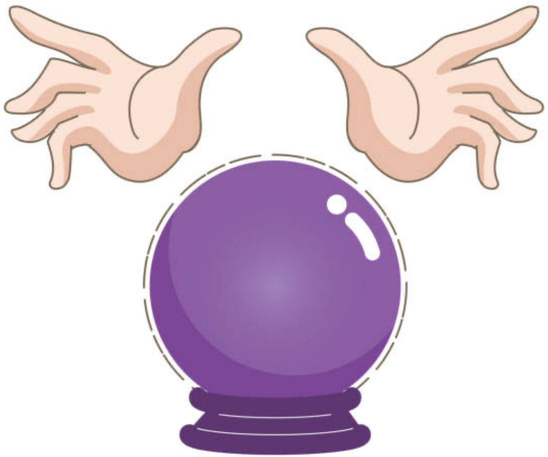

Diagnosis

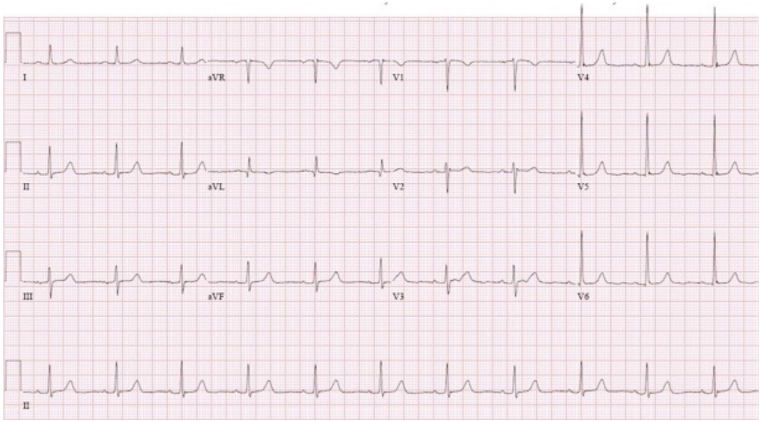

ECG

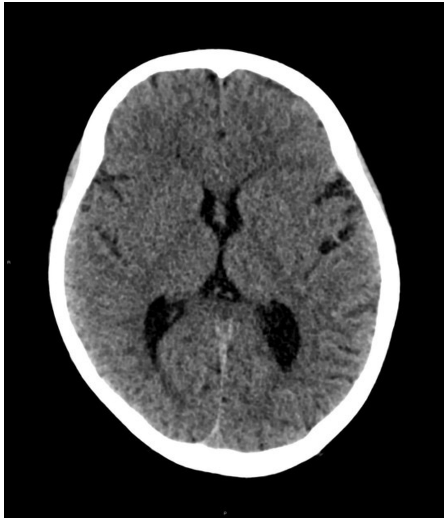

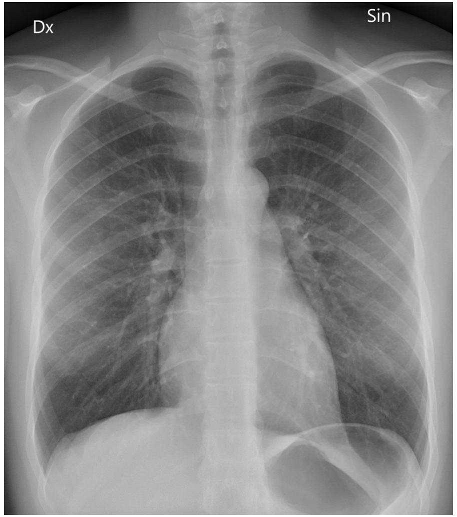

Radiology

Triage

Risk Stratification of Patients

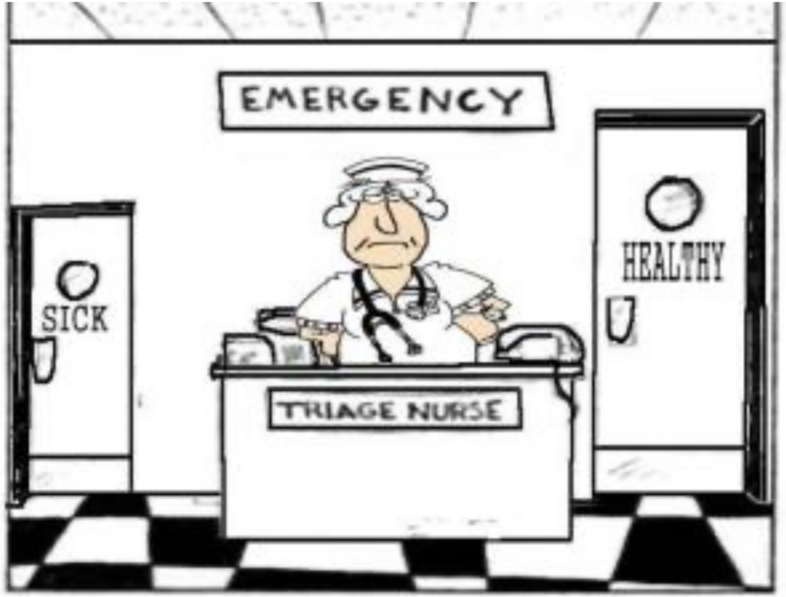

Outline

- Defining AI
- Uses of AI in EM practice
- Current limitations of AI

The Challenges

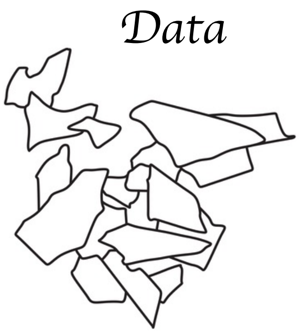

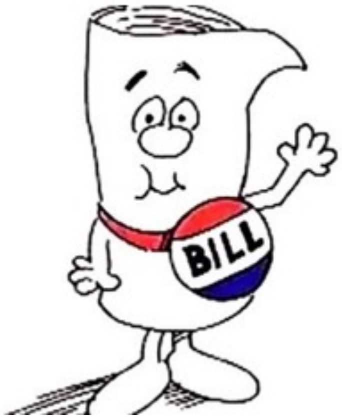

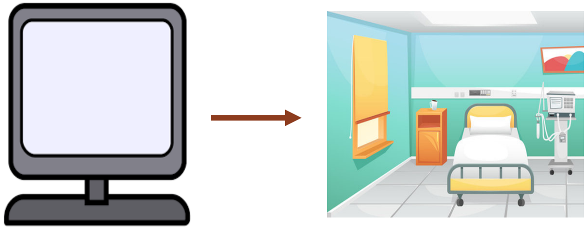

Data Quality

Legal & Regulatory

Implementation

Outline

- Defining AI
- Uses of AI in EM practice
- Current limitations of AI

Augmented Intelligence

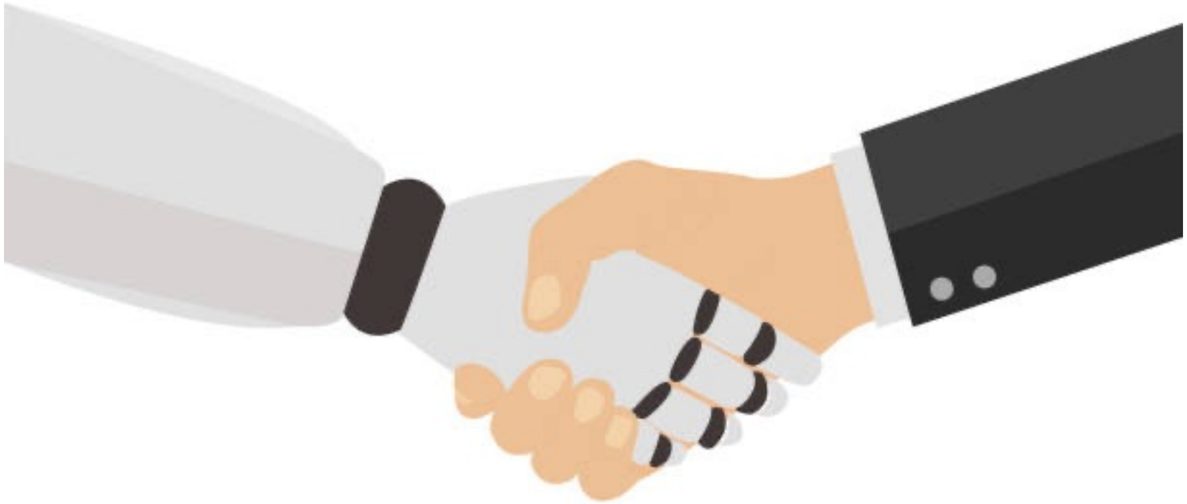

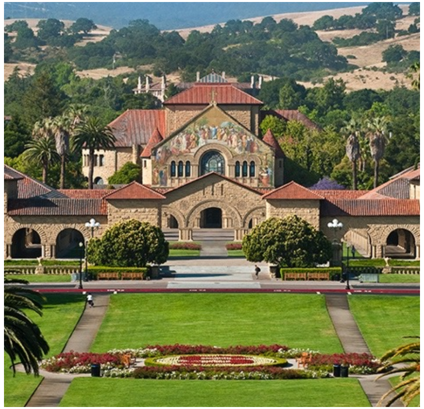

Thank You

gbunney@stanford.edu

Stanford MEDICINE | Emergency Medicine

References

1. Kirubarajan A, Taher A, Khan S, Masood S. Artificial intelligence in emergency medicine: A scoping review. J Am Coll Emerg Physicians Open. 2020 Nov 7;1(6):1691-1702. doi: 10.1002/emp2.12277. PMID: 33392578; PMCID: PMC7771825.
2. Vearrier L, Derse AR, Basford JB, Larkin GL, Moskop JC. Artificial Intelligence in Emergency Medicine: Benefits, Risks, and Recommendations. J Emerg Med. 2022 Apr;62(4):492-499. doi: 10.1016/j.jemermed.2022.01.001. Epub 2022 Feb 11. PMID: 35164977.

Missingness in Medicine

Addressing the messiness of healthcare data

Carl Preiksaitis, MD
Department of Emergency Medicine
Stanford School of Medicine

Stanford MEDICINE | Emergency Medicine

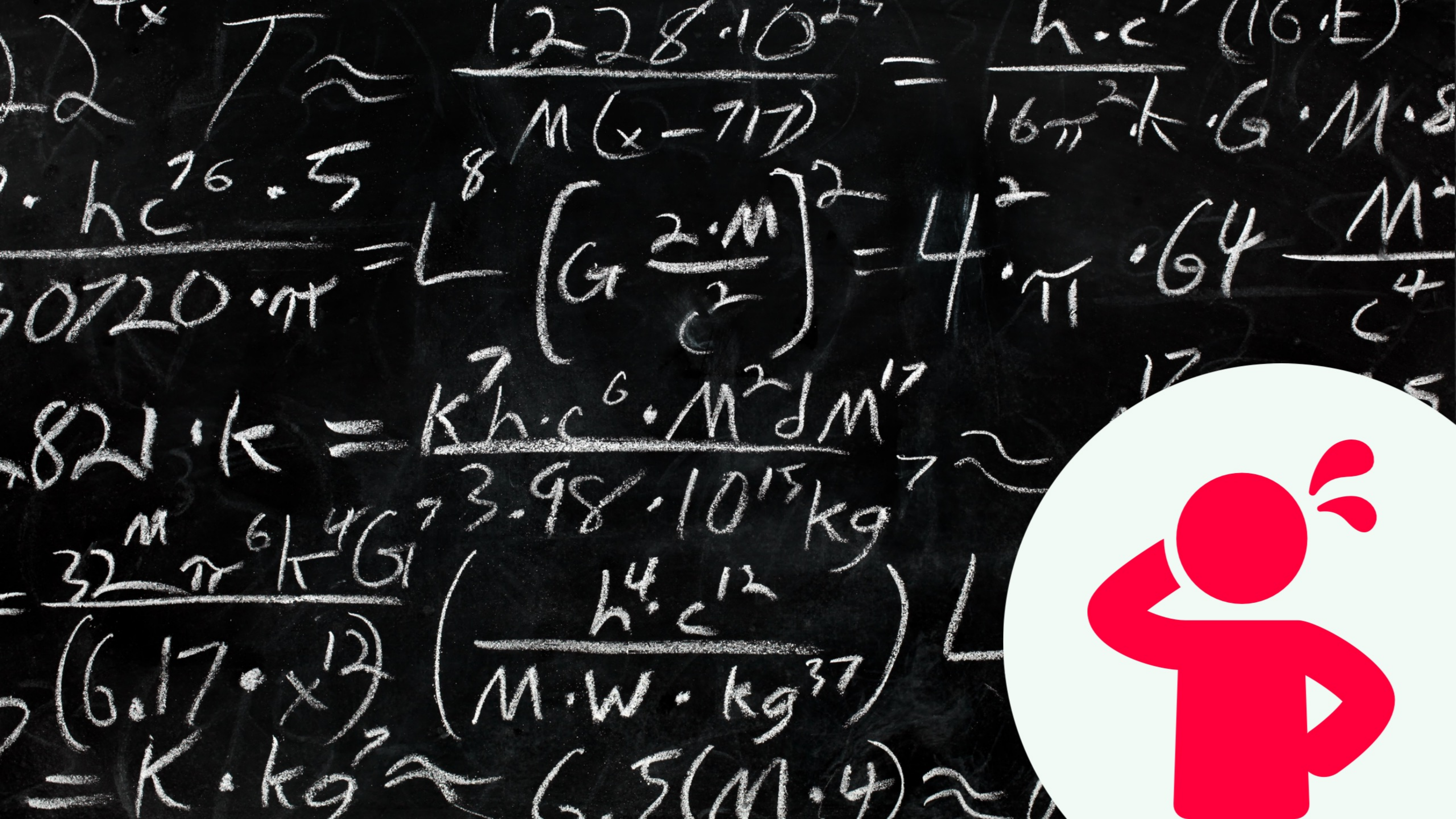

MIA
Missingness in Action

September 22, 2022

A Stanford Conference on the Absence of Data and the Future of AI in Health Care

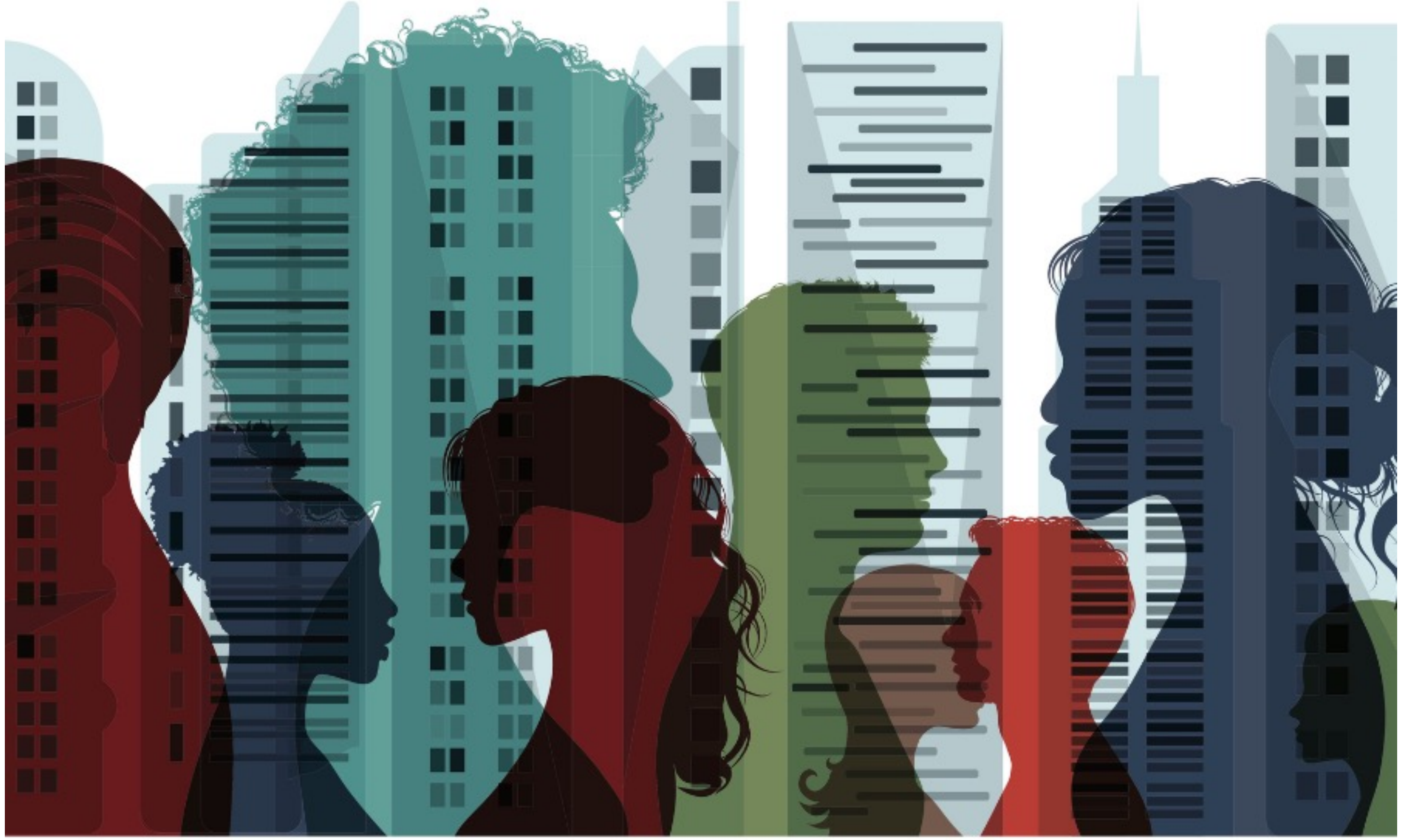

StanfordMIA.org

Stanford MEDICINE | Emergency Medicine

HAI Stanford University Human-Centered Artificial Intelligence

Missingness (n)

The manner in which data are missing from a sample of a population.

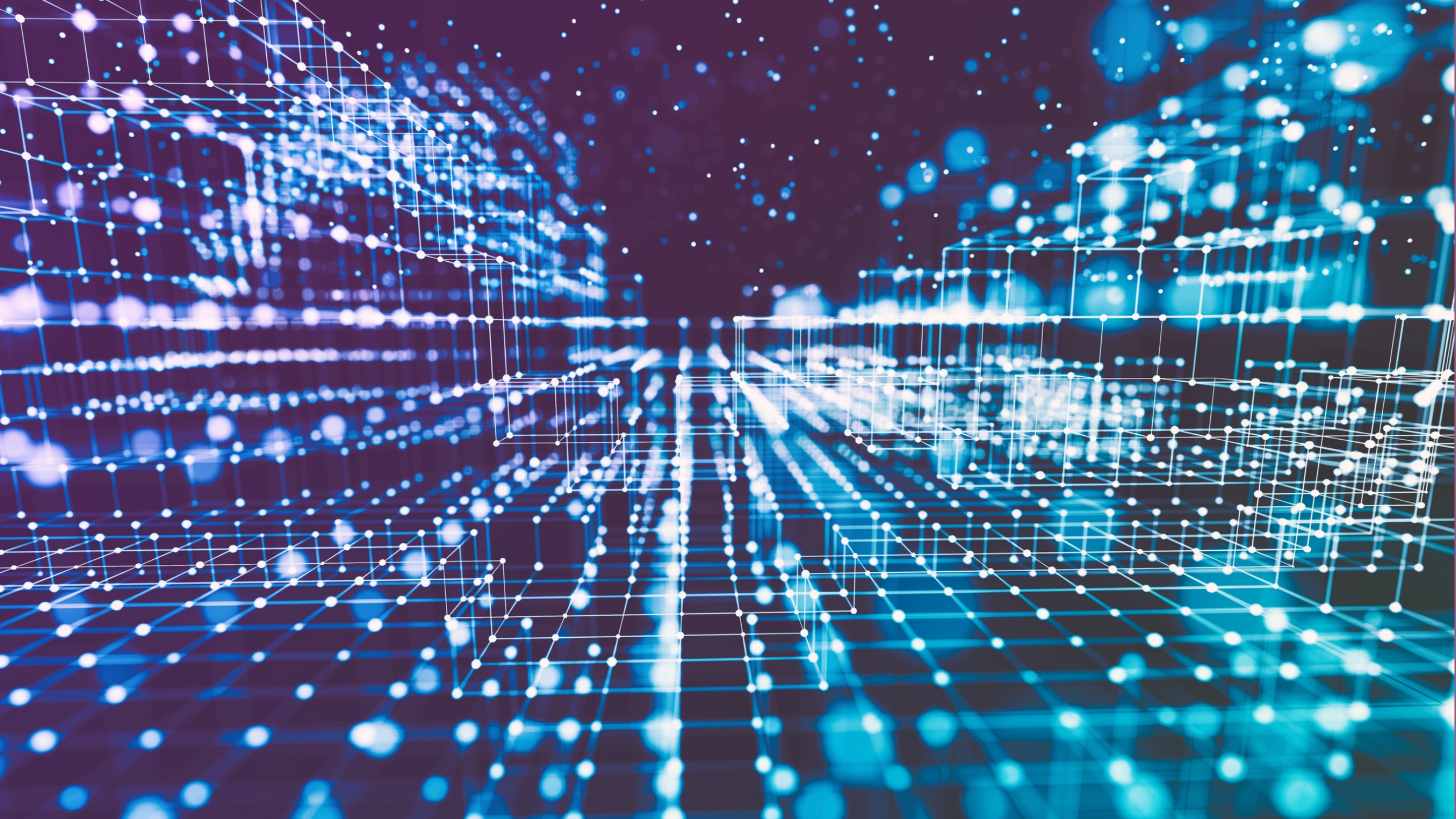

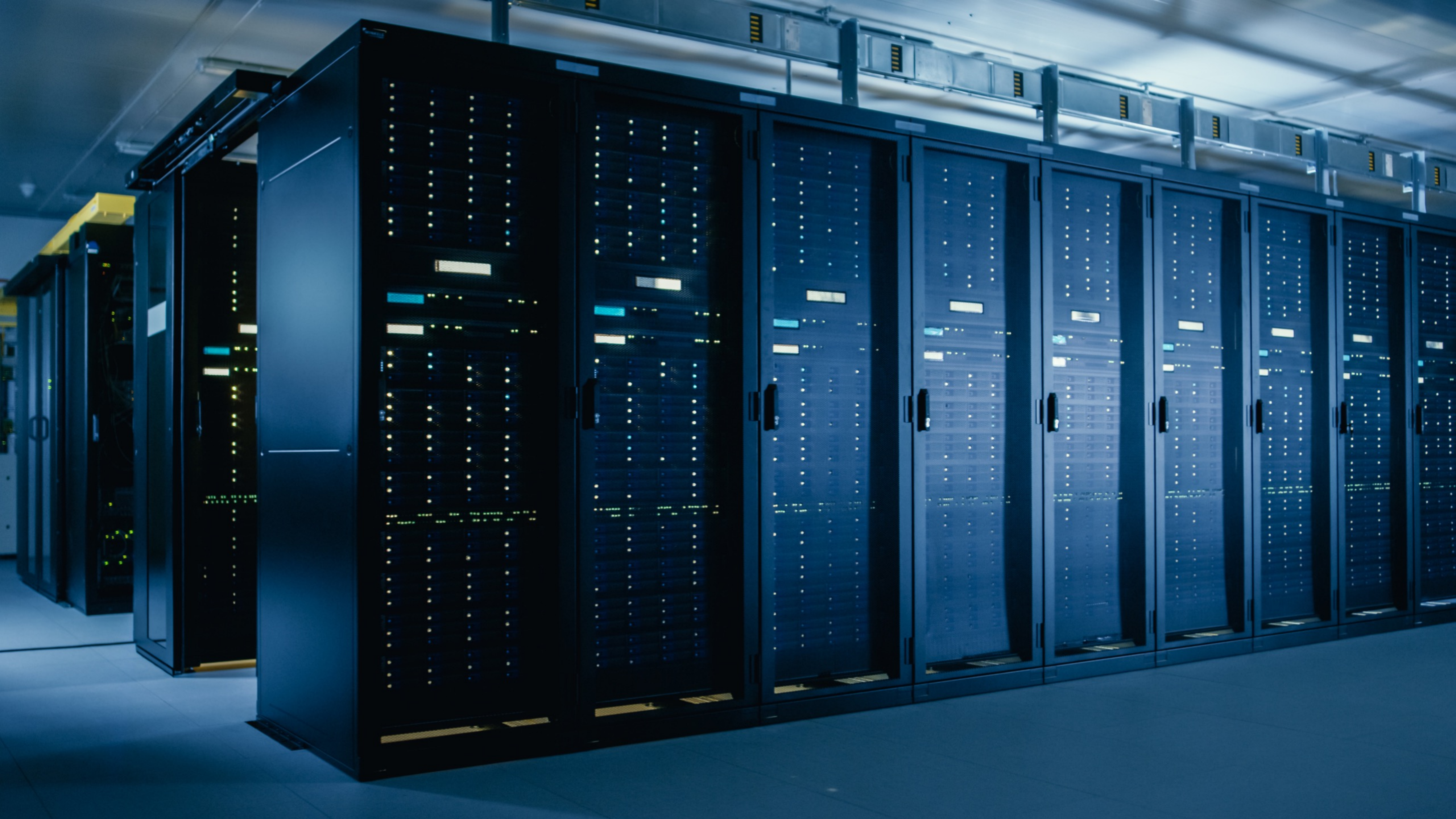

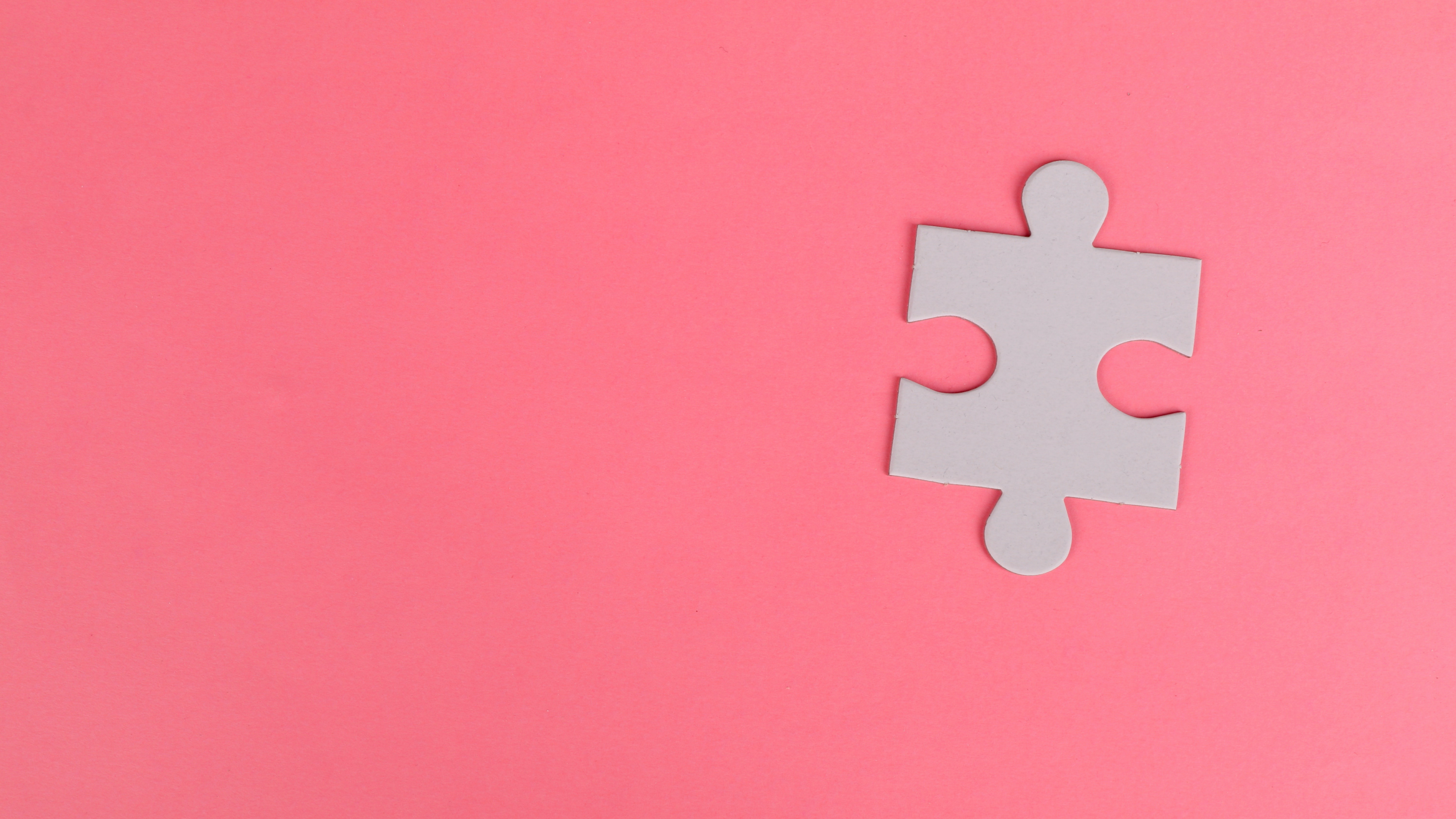

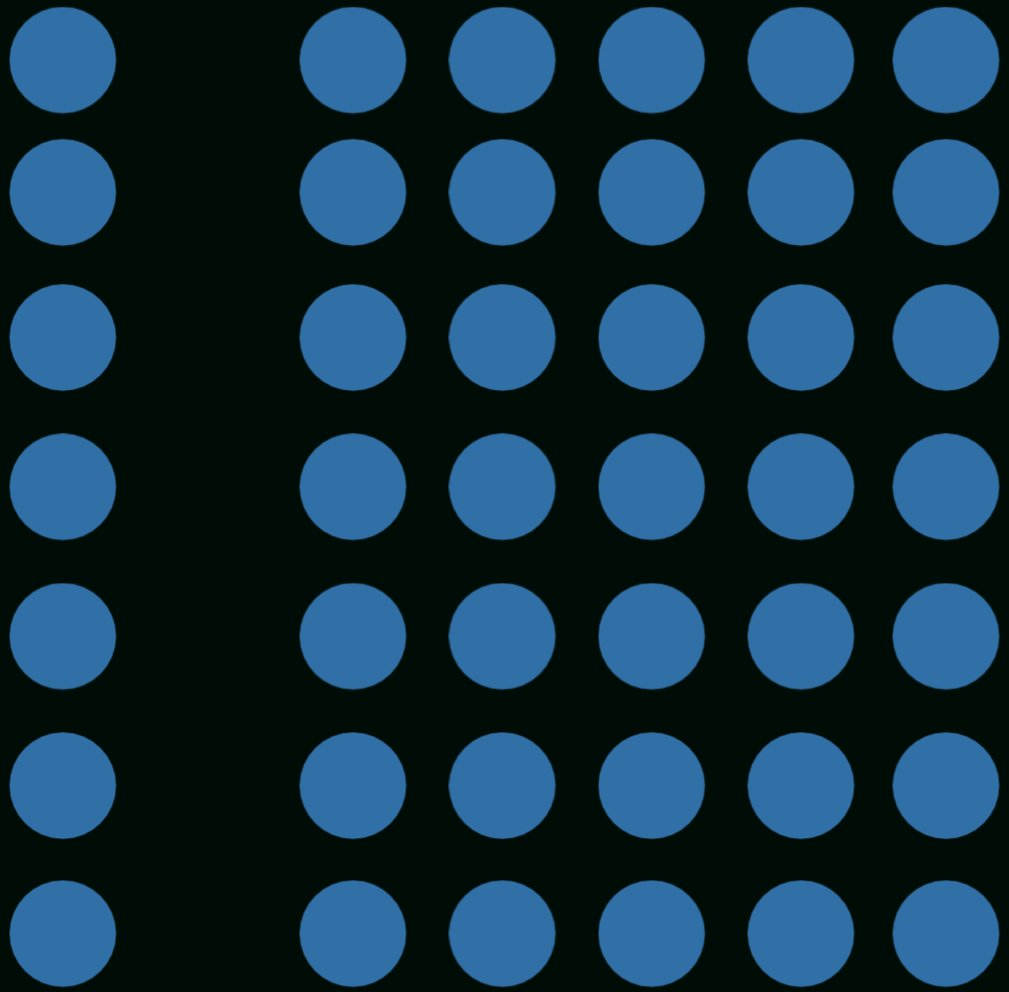

SMD

STRUCTURALLY MISSING DATA

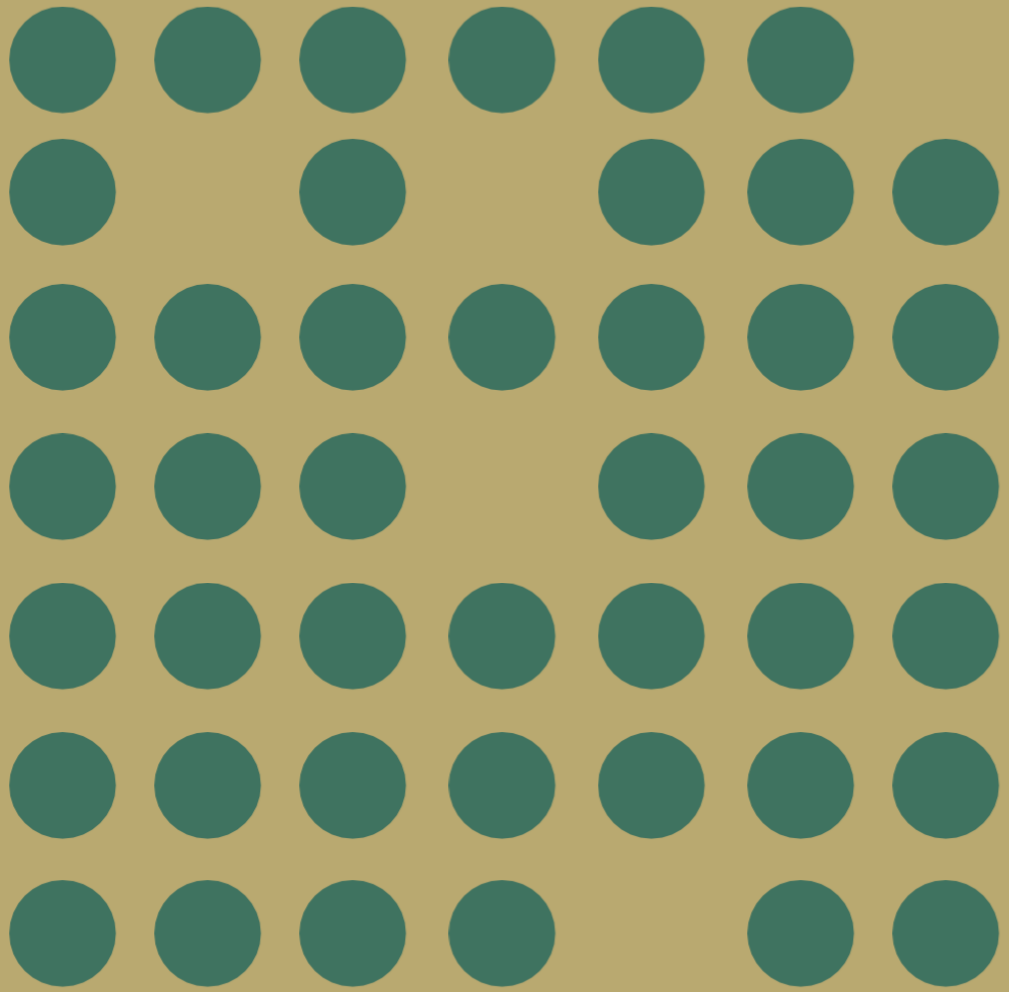

MCAR

MISSING COMPLETELY AT RANDOM

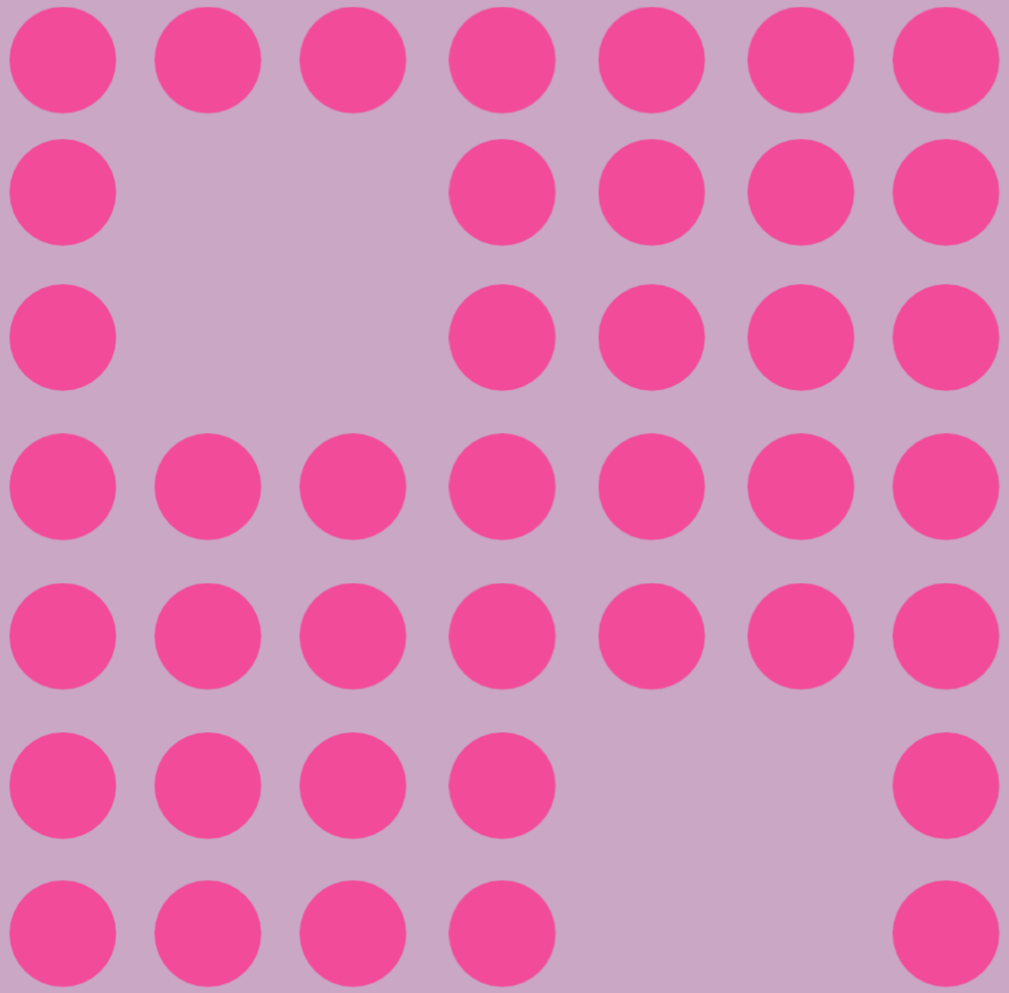

MAR

MISSING AT RANDOM

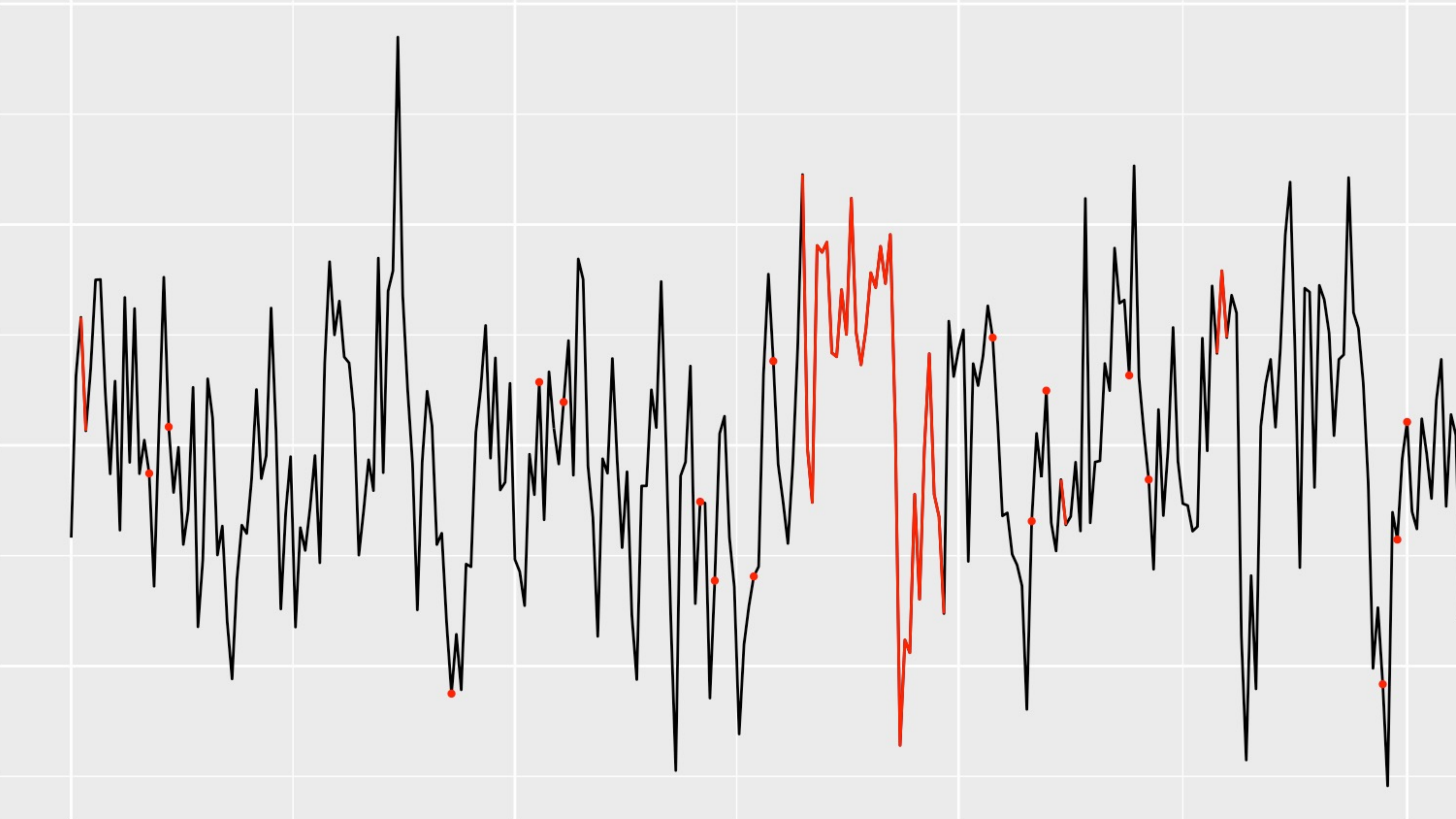

MNAR

MISSING NOT AT RANDOM

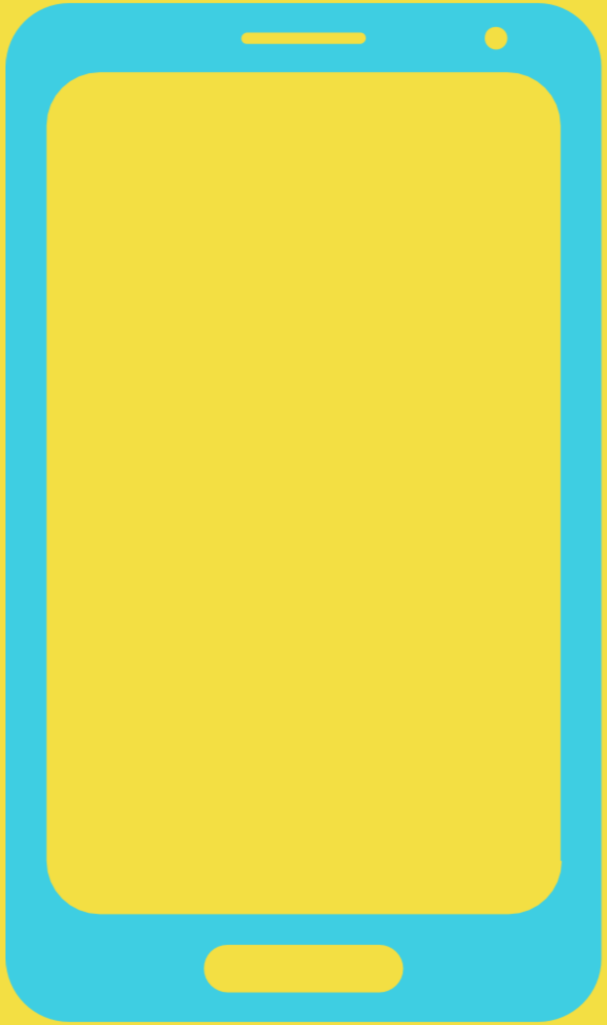

mHealth

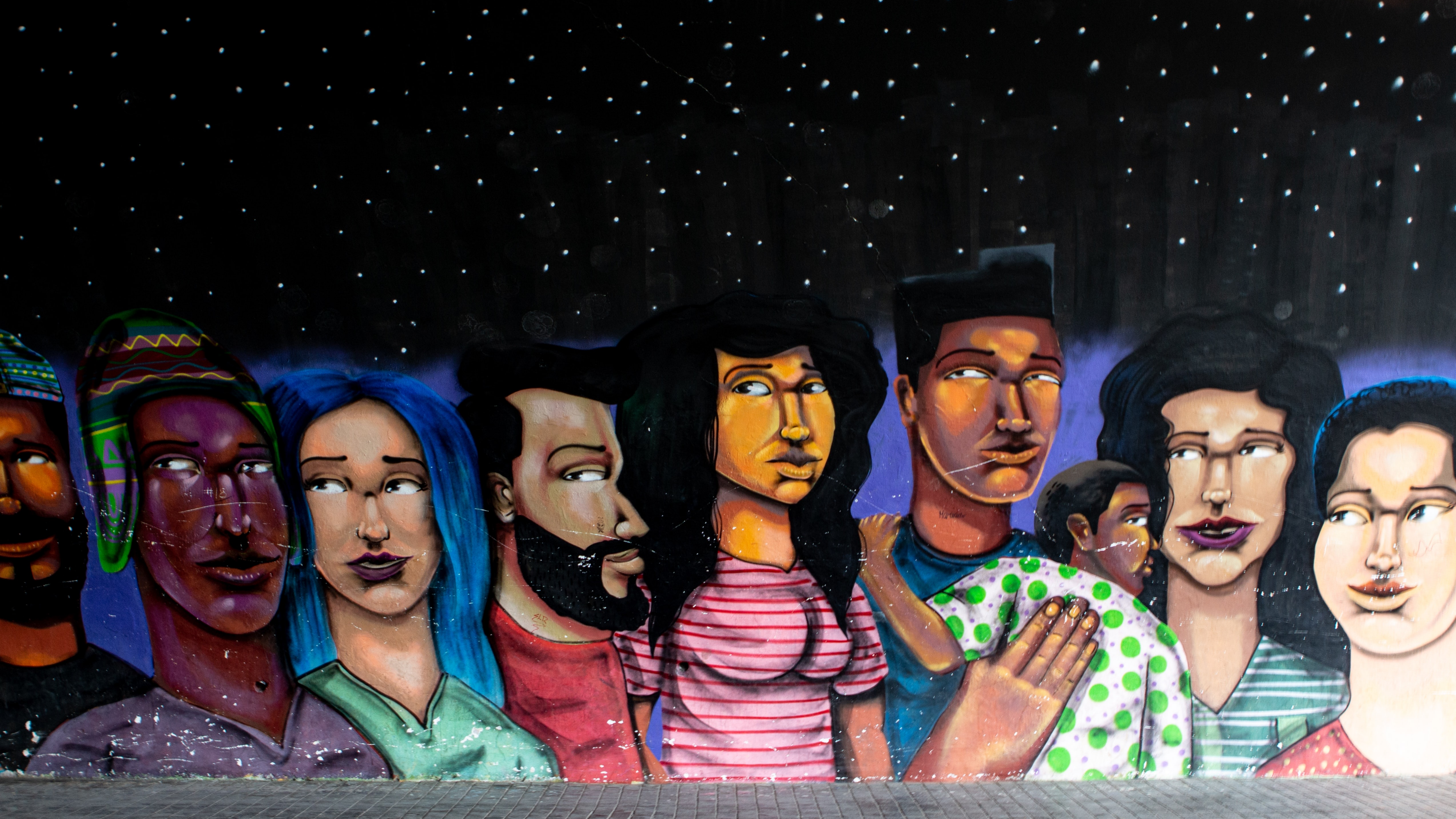

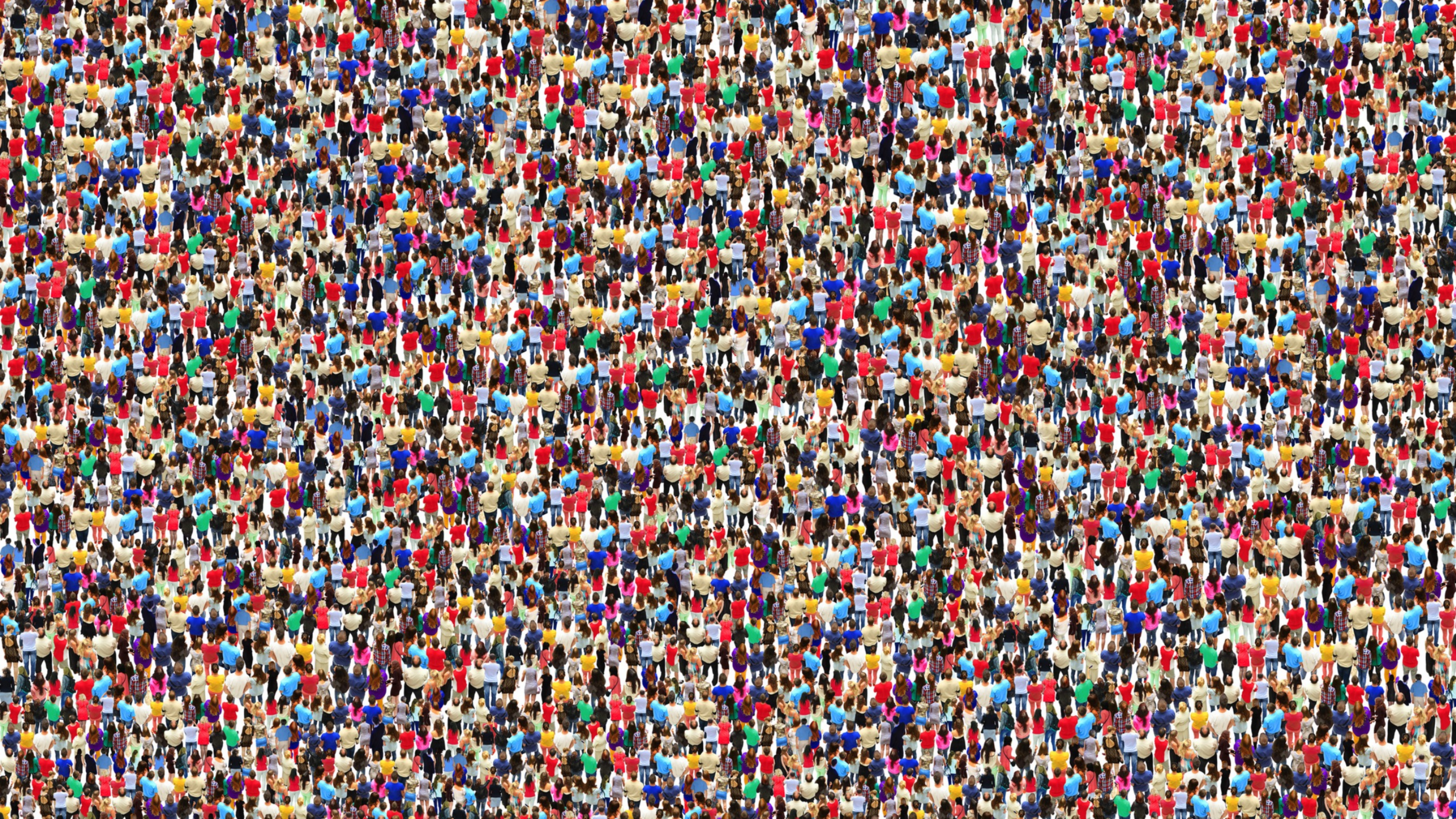

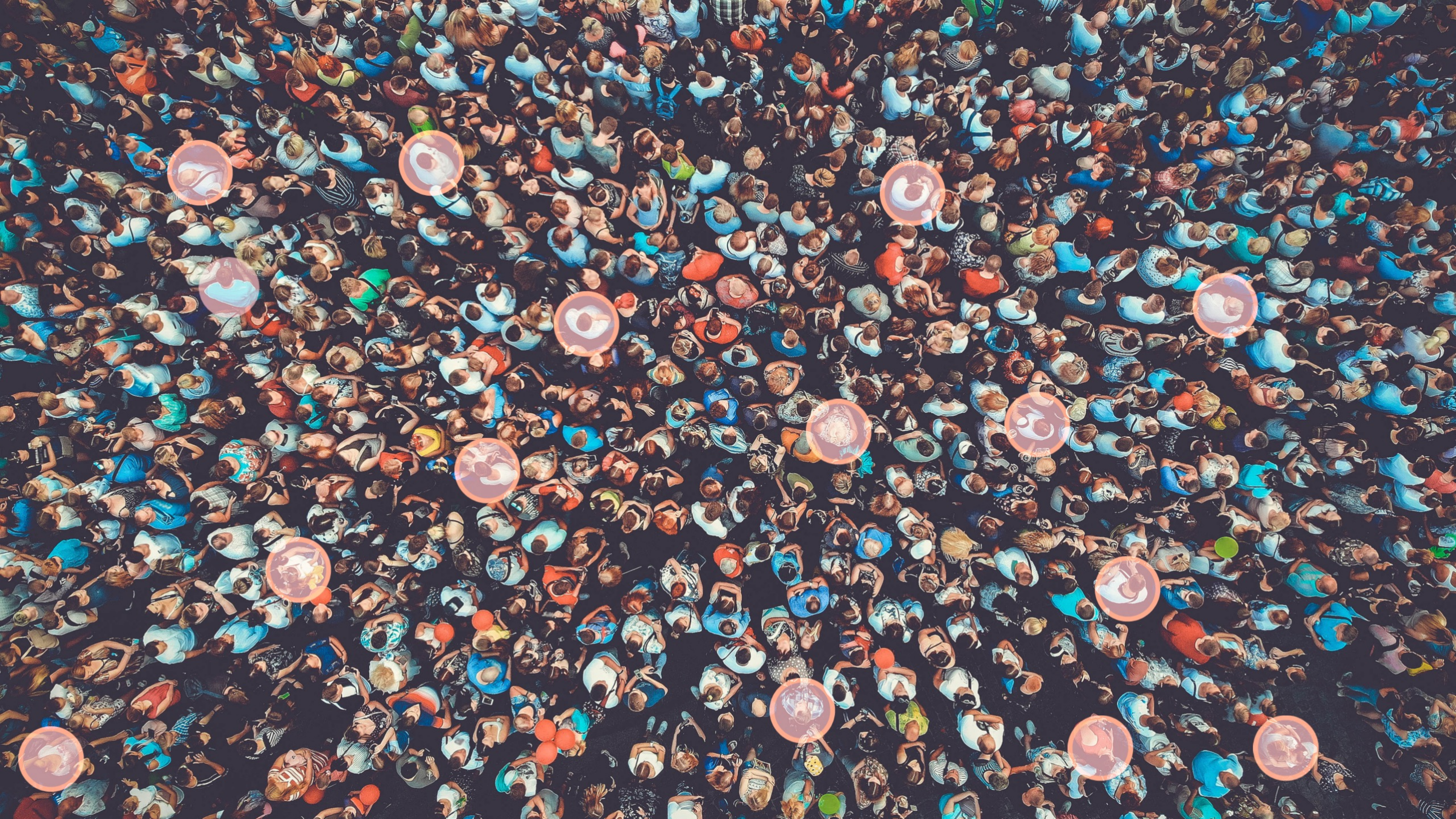

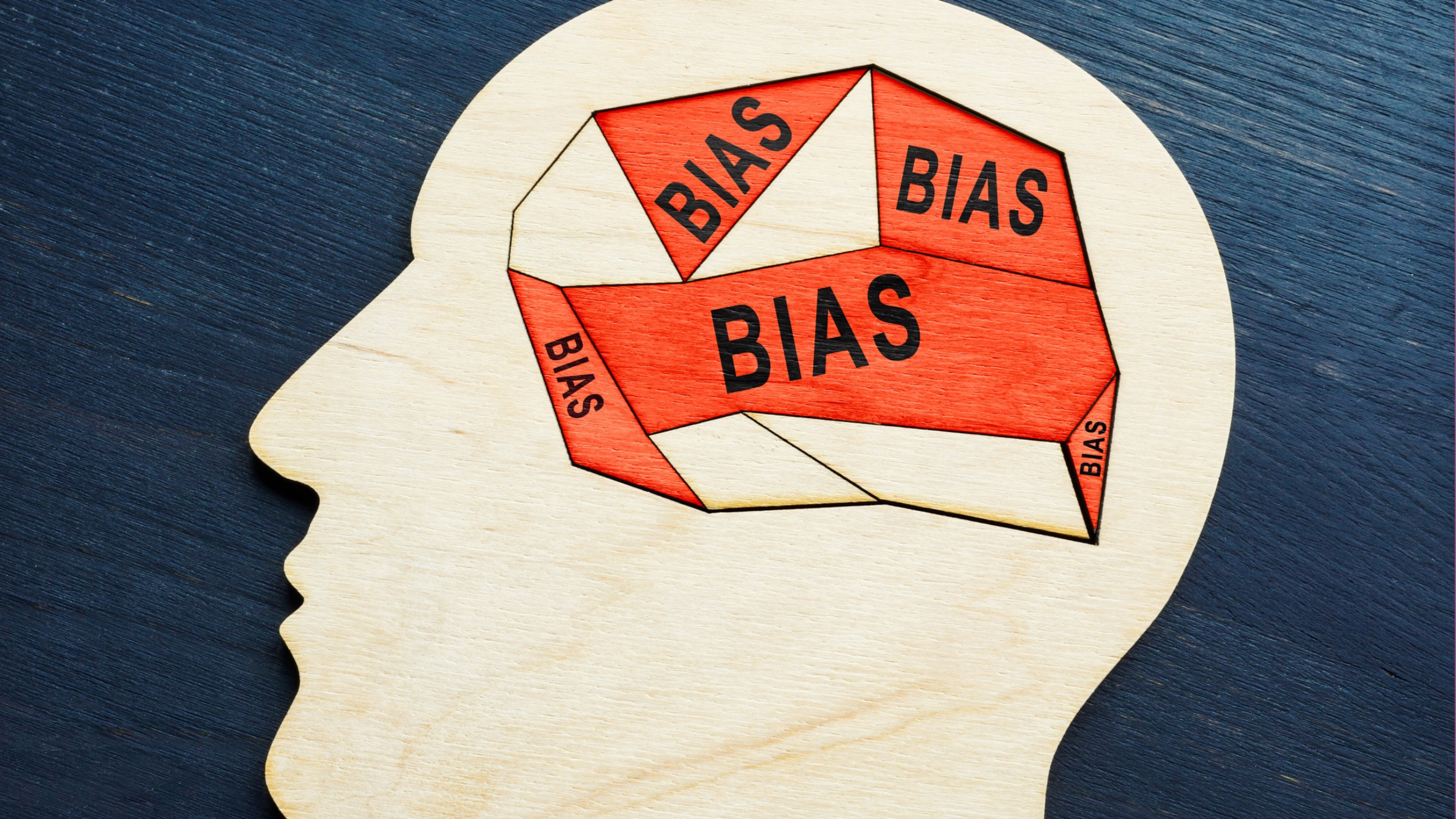
BIAS
BIAS
BIAS
BIAS
BIAS

LABEL CHOICE BIAS

AEA Papers and Proceedings
2021, 111: 37-42
https://doi.org/10.1257/pandp.20211078

On the Inequity of Predicting A While Hoping for B

Sendhil Millainathan and Ziad Obermeyer

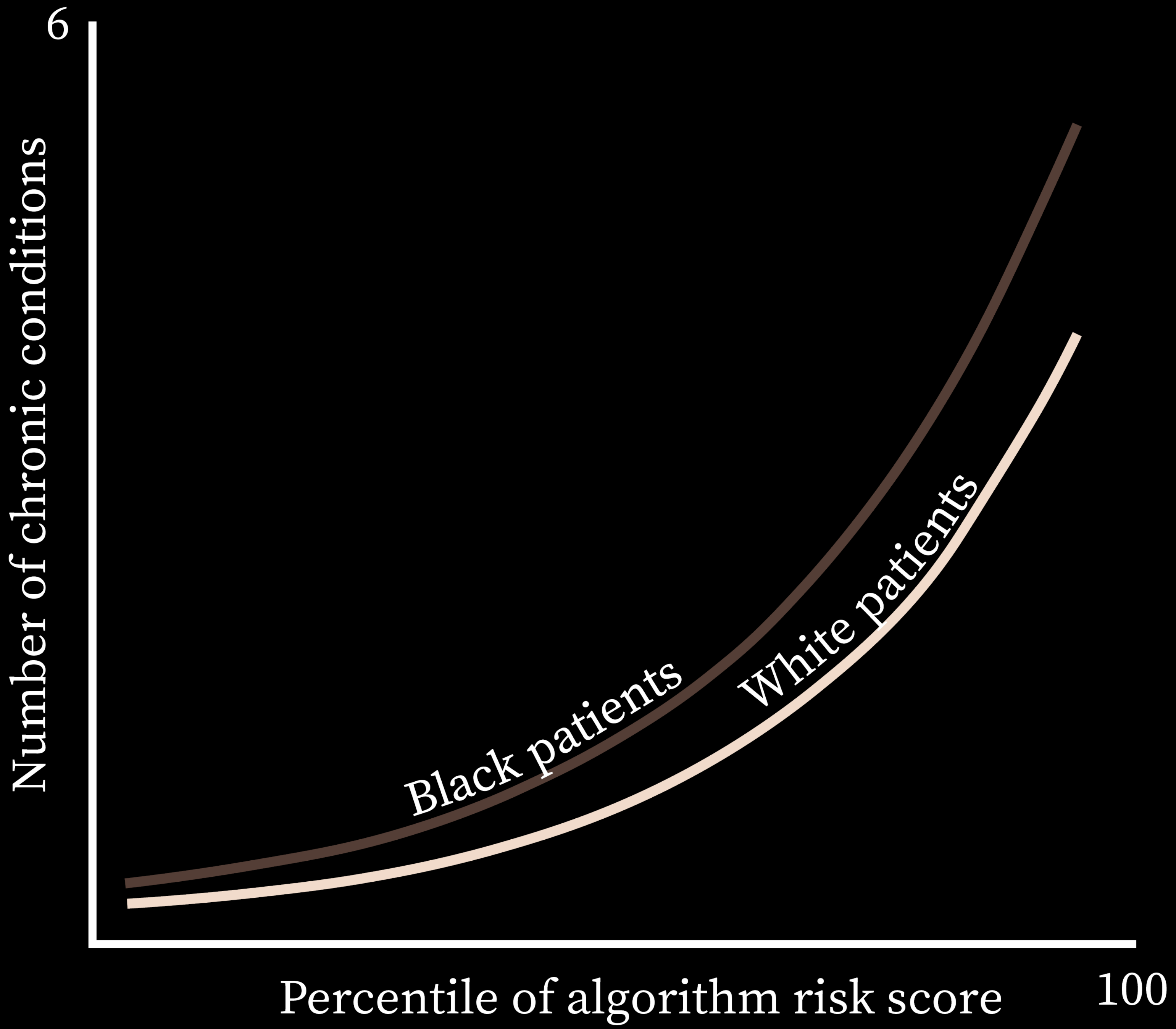
6
Number of chronic conditions
Black patients
White patients
Percentile of algorithm risk score
100

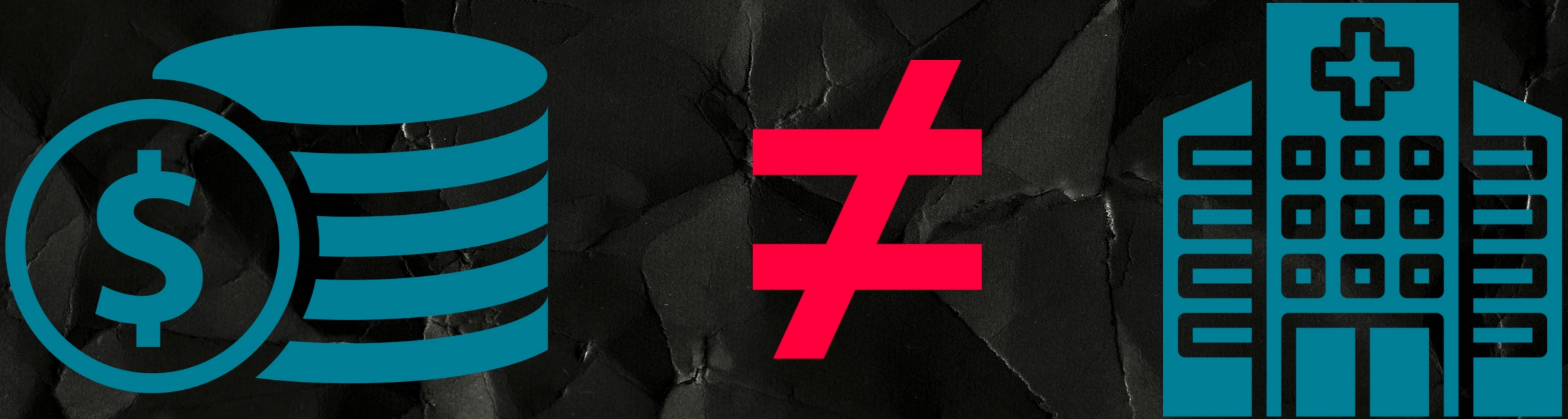

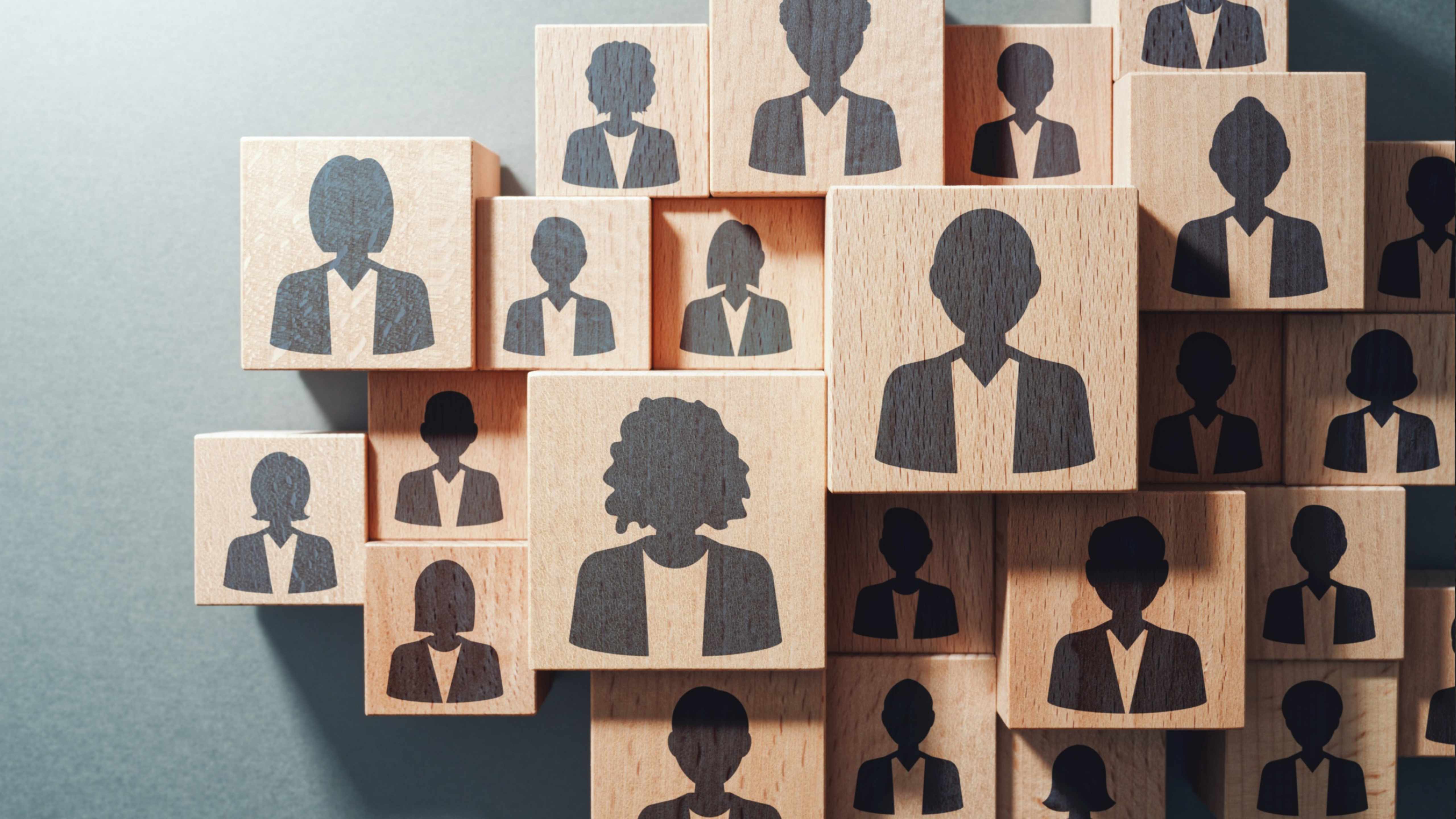

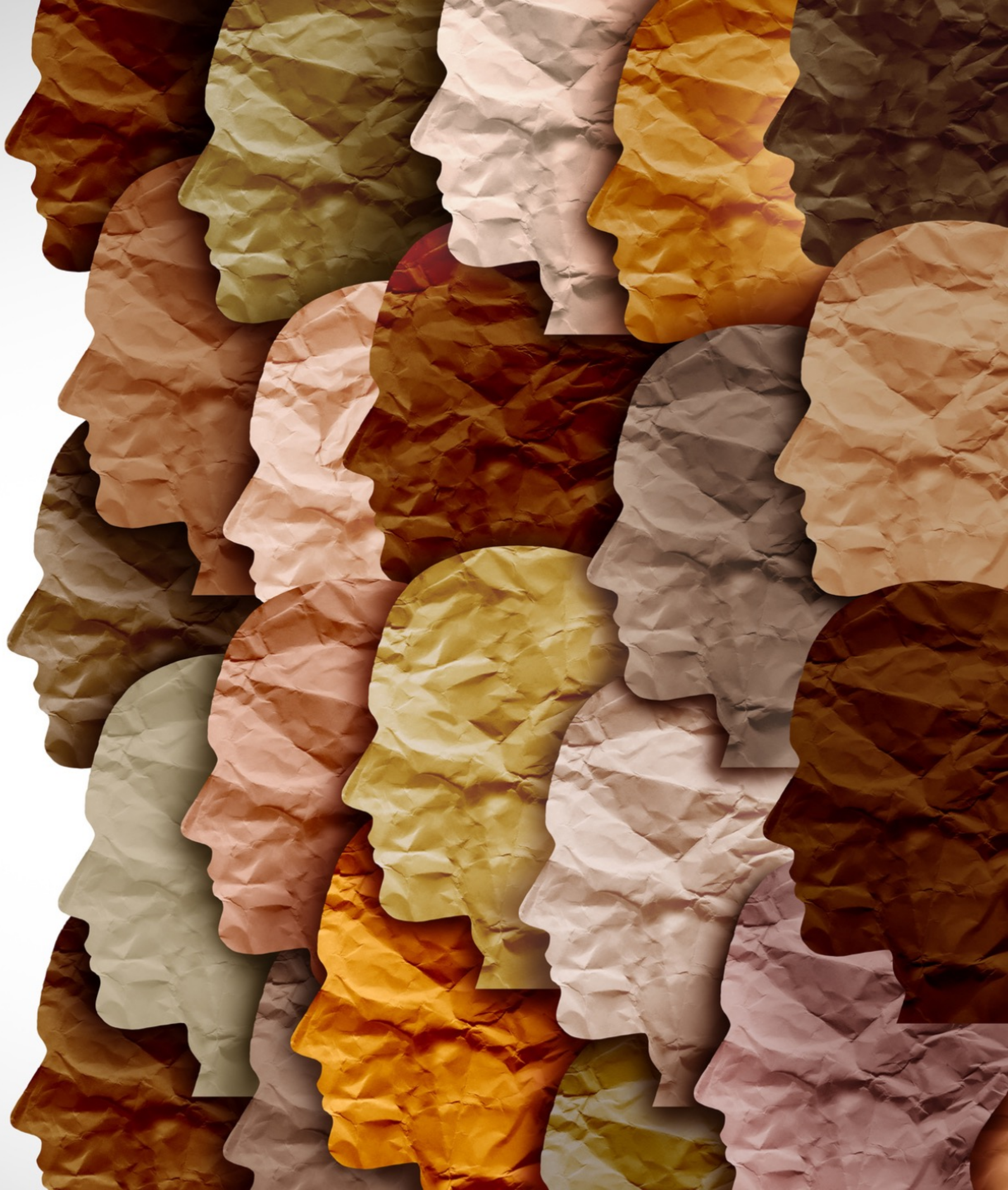

Stanford MEDICINE | Emergency Medicine

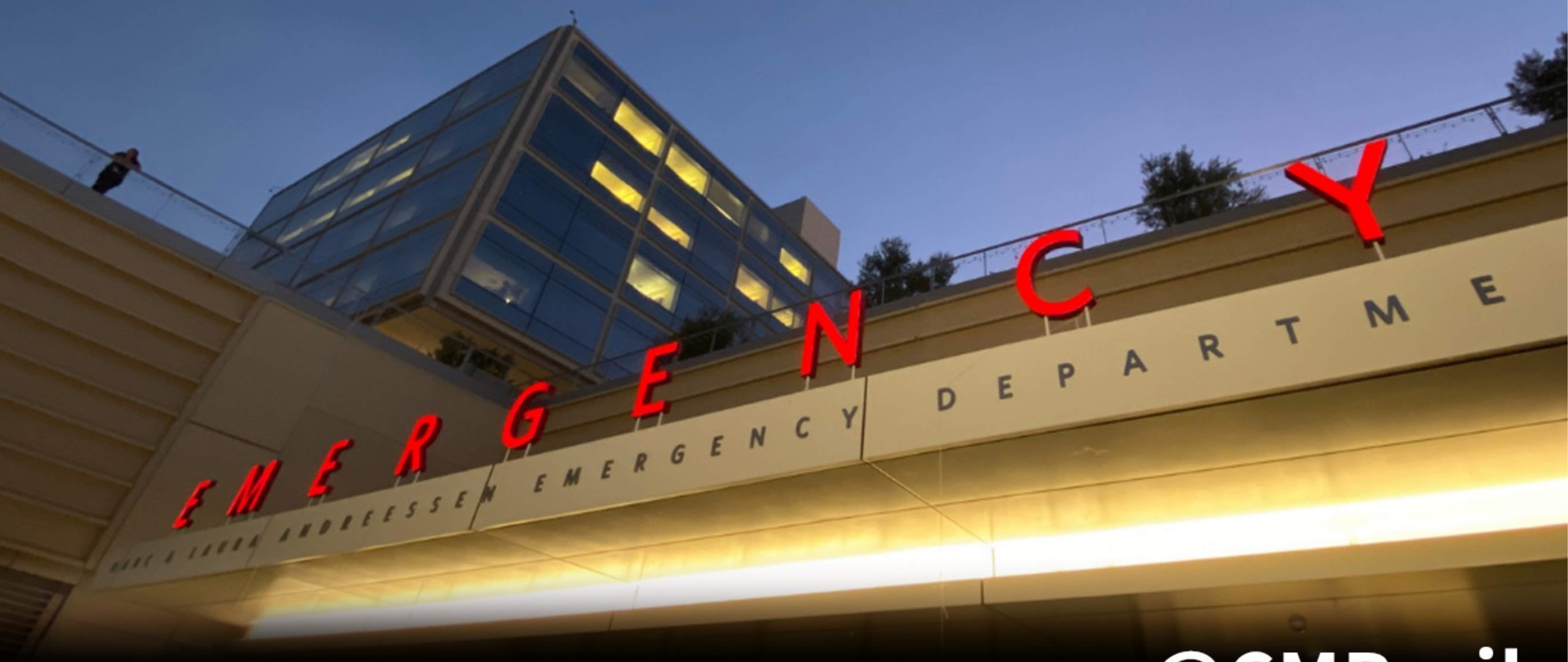

@CMPreik
